# ADAM32 Oncogene in Hepatoblastoma Is Regulated by IGF2BP2

**DOI:** 10.3390/cancers17111772

**Published:** 2025-05-26

**Authors:** Takahiro Fukazawa, Keiji Tanimoto, Masato Kojima, Masami Kanawa, Nobuyuki Hirohashi, Eiso Hiyama

**Affiliations:** 1 Natural Science Center for Basic Research and Development, Hiroshima University, Hiroshima 734-8553, Japan or fukazawa.takahiro.ds@ehime-u.ac.jp (T.F.); mk1019@hiroshima-u.ac.jp (M.K.); mfuku@hiroshima-u.ac.jp (M.K.); 2 Division of Medical Research Support, Advanced Research Support Center, Ehime University, Toon 791-0295, Japan; 3 Department of Radiation Disaster Medicine, Research Institute for Radiation Biology and Medicine, Hiroshima University, Hiroshima 734-8553, Japan; hirohasi@hiroshima-u.ac.jp; 4 Department of Surgery, Graduate School of Biomedical and Health Sciences, Hiroshima University, Hiroshima 734-8551, Japan

**Keywords:** ADAM32, hepatoblastoma, hypoxia, HIF, IGF2BP2

## Abstract

**Background/Objectives**: The membrane protein a disintegrin and metalloproteases (ADAMs) are highly expressed in various human carcinomas and play an important role in cancer characteristics. And among these, ADAM32 is highly expressed in hepatoblastoma (HBL) and plays an important role in oncogenic properties. However, the regulatory mechanism has not been determined. Recently, it has been reported that some ADAMs are regulated by HIF, which is an important transcription factor in response to hypoxia. Therefore, we decided to study the regulatory mechanisms of *ADAM32* under hypoxic conditions by using HBL, breast, and lung cancer cell lines. **Methods/Results**: When these cells were exposed to 1% O_2_ (hypoxia), it was found that the levels of *ADAM32* increased at 48 h in HepG2, MCF7, and MDA-MB-231 but not in HUH-6 or lung cancer lines. However, the promoter activity of the *ADAM32* gene in HepG2 remained unchanged under hypoxic conditions, suggesting that the level of *ADAM32* in HBL is regulated by factors other than the promoter activity. From the microarray data, we found that the level of *IGF2BP2*, which is an m^6^A-related molecule, correlated with that of *ADAM32*, and these levels were decreased by *HIF1A* knockdown. And *IGF2BP2* knockdown decreased the expression of ADAM32 and attenuated the increased expression of ADAM32 under hypoxic conditions. **Conclusions**: This study demonstrated that the oncogenic gene *ADAM32* is regulated by IGF2BP2 and that IGF2BP2 could be a molecular target for HBL anticancer therapy.

## Simple Summary

Since hepatoblastoma (HBL) is a hepatic malignancy, part of which is still resistant to anticancer drug treatment, more effective therapy is desired to be developed. Our previous study showed that ADAM32 is highly expressed in HBL and plays an important role in the oncogenic property. However, the regulatory mechanisms were not determined. In this study, we focused on hypoxia, which is a characteristic of the cancer microenvironment. Then, we demonstrated that the expression levels of *ADAM32* increased under hypoxic conditions, and these expressions are regulated by IGF2BP2. Thus, our study suggested that IGF2BP2 could be a molecular target for anticancer therapy of HBL.

## 1. Introduction

Hepatoblastoma (HBL) is the most prevalent malignant hepatic neoplasm in children, accounting for approximately 80% of all pediatric hepatic malignancies [1,2,3,4]. Its incidence has been rising worldwide, particularly in preterm and low-birthweight infants, suggesting a complex interplay between genetic predisposition and environmental factors [5]. Despite significant advances in multimodal therapy, including surgical resection, chemotherapy, and liver transplantation, outcomes for advanced or recurrent cases remain suboptimal. The standard chemotherapeutic regimen for HBL is primarily based on cis-diamminedichloroplatinum (CDDP), which, while highly effective, is associated with severe dose-limiting toxicities such as ototoxicity, nephrotoxicity, and an increased risk of secondary leukemia [1,2,3,4]. These challenges underscore the urgent need for safer and more effective therapeutic strategies targeting key molecular pathways that drive tumor progression.

Among these pathways, the a disintegrin and metalloproteinase (ADAM) and a disintegrin and metalloproteinase with thrombospondin motifs (ADAMTS) families have emerged as critical regulators of tumorigenesis [6,7,8]. These transmembrane and secreted proteases play essential roles in cell adhesion, migration, extracellular matrix remodeling, and the proteolytic cleavage of membrane-bound proteins, all of which contribute to cancer progression [6,7,8]. Notably, several members of the ADAM and ADAMTS families have been implicated in critical processes such as angiogenesis, metastasis, and chemoresistance, underscoring their potential as viable therapeutic targets [6,7,8]. Of particular note is ADAM32, which has attracted attention due to its upregulation in various cancers, including HBL. Our previous studies have shown that ADAM32 expression is high and plays a crucial oncogenic role in HBL, driving tumor cell proliferation, migration, and resistance to chemotherapy [9]. Notably, the modulation of ADAM32 expression has been shown to sensitize HBL cells to CDDP, while its upregulation has been associated with increased cell motility and cancer stemness. These findings suggest that ADAM32 may be a valuable target for combination therapy with standard chemotherapy. However, the mechanisms regulating ADAM32 expression in HBL remain to be fully elucidated.

Hypoxia, a hallmark of rapidly growing solid tumors, is one of the best-characterized regulatory factors in the tumor microenvironment. Hypoxia-inducible factors (HIFs) function as central mediators of the cellular response to low oxygen levels, orchestrating the expression of genes involved in angiogenesis, glucose metabolism, epithelial–mesenchymal transition, and cell survival [10,11,12]. Several ADAM and ADAMTS proteins have been identified as direct transcriptional targets of HIFs, thereby promoting increased invasiveness and therapy resistance under hypoxic conditions [13,14]. Given that HBL exists in a hypoxic microenvironment, it is plausible that ADAM32 expression is regulated by HIF-dependent mechanisms. HIFs have been shown to regulate hypoxia-induced gene expression primarily by binding to hypoxia response elements (HREs) in the promoter regions of target genes [15,16]. However, whether ADAM32 is a hypoxia-responsive gene remains to be elucidated. While HIFs are predominantly known as transcriptional regulators, recent studies suggest that post-transcriptional mechanisms also play a crucial role in fine-tuning gene expression under hypoxic conditions. One such mechanism involves N6-methyladenosine (m^6^A) modification, the most prevalent internal RNA modification in eukaryotic mRNA, which affects mRNA stability, splicing, translation, and degradation [17]. It has been reported that m^6^A modification plays an important role in cancer progression. And dysregulated m^6^A-related genes, such as fat mass and obesity-associated protein (FTO), methyltransferase 3, N6-adenosine methyltransferase complex catalytic subunit (METTL3), and insulin-like growth factor 2 mRNA binding proteins (IGF2BPs) contribute to cancer progression, metastasis, and metabolism by regulating oncogenes [18]. Despite mounting evidence for the pivotal role of m^6^A modification in cancer biology, its potential role in regulating ADAM32 expression under hypoxic conditions remains to be elucidated.

To address these knowledge gaps, we aim to investigate the regulatory mechanisms of ADAM32 expression in HBL, focusing on both hypoxia-driven transcriptional control and post-transcriptional RNA modifications. A comprehensive understanding of these mechanisms may provide novel insights into the upstream regulators of ADAM32 and facilitate the identification of novel therapeutic targets that could improve the efficacy of HBL treatment outcomes.

## 2. Materials and Methods

### 2.1. Plasmid Construction

To evaluate the promoter activity of ADAM32, the 5′ promoter and the adjacent downstream region of *ADAM32* were amplified from human genomic DNA by PCR using Fast Start Taq DNA Polymerase (Roche, Basel, Switzerland). The amplified fragments were subsequently cloned into pcDNA3.1/V5-His (Invitrogen, Carlsbad, CA, USA), followed by subcloning into pGL4.26 (Promega Corporation, Madison, WI, USA). Five reporters were constructed within the regions −2191 to +245 (rep −2 kb), +243 to +1548 (rep #1), +1974 to +2468 (rep #2), +5210 to +6176 (rep #3), and +6630 to +7152 (rep #4) (Figure S1A,B). For the stable knockdown experiment, pSUPERIOR-puro (OligoEngine, Seattle, WA, USA) was utilized. The target sequences of short hairpin RNA (shRNA) were designed using siDirect version 2 (http://sidirect2.rnai.jp, accessed on 29 July 2021) in accordance with a previously described protocol [19,20]. Subsequently, the oligonucleotides for the target gene were annealed and subcloned into the pSUPERIOR-puro vector. The shRNA target sequences utilized in these cloning procedures are enumerated in Table S1. Constructs were verified by sequence analysis. The shRNA vector targeting *lacZ* was designated as shLacZ and insulin-like growth factor 2 mRNA binding protein 2 (*IGF2BP2*) as shIGF2BP2.

### 2.2. Cell Culture

The HBL cell line, HepG2 derived from poorly differentiated epithelial HBL (American Type Culture Collection, Manassas, VI, USA; ATCC) and HUH-6 derived from well differentiated epithelial HBL (Japanese Collection of Research Bioresources Cell Bank), breast cancer cell line, MCF7, MDA-MB-231, lung cancer cell line, A549, and PC6 (ATCC) were seeded on 100 mm culture dishes in RPMI (NACALAI TESQUE, Inc., Kyoto, Japan) containing 10% FBS (BioWhittaker, Verviers, Belgium) and 100 μg/mL kanamycin (Sigma, St. Louis, MO, USA) [21,22]. Cultures were maintained at 37 °C in 5% CO_2_. For the transient knockdown experiment, AllStar Negative control siRNA (1027281, Qiagen, Inc., Valencia, CA, USA) abbreviated as siN or siRNA for *HIF1A* (SI02664053, Qiagen), abbreviated as siHIF1A, was transfected into HepG2 cells using Lipofectamine RNAiMAX (Thermo Fisher Scientific K.K., Tokyo, Japan). The cells were then exposed to normoxia or hypoxia for 48 h. Stable cell lines for *IGF2BP2* knockdown were established by transfection of shLacZ for control or shIGF2BP2 for knockdown experiments according to the manufacturer’s protocol. HepG2 cells stably expressing shLacZ and shIGF2BP2 were designated as HepG2 shLacZ and HepG2 shIGF2BP2, respectively.

### 2.3. Real-Time RT-PCR

Total RNA was extracted using NucleoSpin^®^ RNA (MACHEREY-NAGEL GmbH&Co., KG, Düren, Germany) according to the manufacturer’s protocol, and cDNA was synthesized using a High-Capacity cDNA Reverse Transcription Kit (Applied Biosystems, Foster City, CA, USA). Real-time RT-PCR was performed using a 7900HT or 7500 (Applied Biosystems) and FastStart Universal Probe Master (Roche) according to the TaqMan probe method. *ACTB* (4326315E, Applied Biosystems) or *18S* (4310893E, Applied Biosystems) was used as an internal control (Figure S2). Primer and probe sets are listed in Table S2.

### 2.4. Immunoblotting

Proteins were extracted from whole cell pellets as previously described [23]. Twenty to forty micrograms of each protein sample were resolved on a 5–12% gradient sodium dodecyl sulfate-polyacrylamide gel (ATTO, Tokyo, Japan) and blotted onto PVDF membranes (Millipore, Burlington, MA, USA). After blocking with 2% BSA or 5% skim milk in TBS for 1 h at room temperature, the membranes were incubated overnight at 4 °C with primary antibodies diluted in CanGet signal primary buffer (TOYOBO, Osaka, Japan). After washing with TBST, the membranes were incubated with horseradish peroxidase-linked anti-rabbit IgG (Cell Signaling TECHNOLOGY, Danvers, MA, USA; CST) or anti-mouse IgG (CST) diluted in CanGet signal secondary antibody buffer for 1 h at room temperature. After washing with TBST, the membranes were incubated with Pierce Western Blotting Substrate Femto (Pierce, Rockford, IL, USA) and developed on X-ray film (Fujifilm, Tokyo, Japan). Primary antibodies and conditions were as follows: anti-ADAM32 (HPA044156; Sigma) at 1:500; anti-HIF-1α (610959; BD Pharmingen, San Diego, CA, USA) at 1:1000; anti-IGF2BP2 (RN008P; MBL) at 1:2000; anti-β-actin (A5316; Sigma) at 1:5000. Experiments were performed with at least three independent replicates.

### 2.5. Luciferase Reporter Assays

The luciferase reporter vector was constructed as described previously. HepG2 cells were seeded in 24-well plates and cultured for one day. The reporter constructs rep −2 kb, rep #1, rep #2, rep #3, and rep #4 were transiently co-transfected into HepG2 cells using TransIT-LT1 Transfection Reagent (Mirus Bio LLC, Madison, WI, USA). After 4 h, the transfected cells were exposed to 20% O_2_ (normoxia) or 1% O_2_ (hypoxia). For the overexpression of HIF1α, p3xFLAG-HIF1α was used as previously described [24]. The Renilla luciferase vector (pRL-SV40, Promega) was used to assess transfection efficiency. After 48 h of hypoxia, luminescence was assessed using a Biolumat LB 9505 luminometer (Berthold Technologies, Bad Wildbad, Germany) with the Dual-Luciferase Reporter Assay System (Promega). Relative luciferase activities (RLAs) are reported as the ratio of firefly/renilla luciferase activities, and the average of at least three independent experiments was calculated.

### 2.6. Bioinformatics Analysis

The m^6^A sites of *ADAM32* mRNA were predicted using the web-based application SRAMP (sequence-based RNA adenosine methylation site predictor) (http://www.cuilab.cn/sramp, accessed on 7 April 2020) [25]. And the sites with very high confidence by SRAMP were then selected as the m^6^A candidate site (Table S3). A DNA microarray dataset (GSE131329) containing expression data of HBL (14 paired tumor and non-cancerous liver tissue samples) was obtained from the Gene Expression Omnibus and analyzed using GEO2R to determine differentially expressed genes (DEGs). DEGs were defined as those with a log fold change greater than 1.0 and Benjamini–Hochberg false discovery rate with *p* values < 0.05.

### 2.7. SELECT Assay

A single-base elongation- and ligation-based qPCR amplification method (SELECT) was performed as previously described [26,27]. Briefly, total RNA was mixed with 40 nM up primer, 40 nM down primer and 5 μM dNTP (TOYOBO) in 13 μL 1×CutSmart buffer (B7204S; NEB, Ipswich, MA, USA). The RNA and primers were annealed by temperature gradient (1 min each at 90 °C, 80 °C, 70 °C, 60 °C, 50 °C, and 40 °C for 6 min). Next, 2 μL of a mixture containing 0.01 U Bst 2.0 DNA polymerase (M0537S; NEB) and 10 nmol dATP (4026; Takara Bio, Shiga, Japan) was added to 13 μL of the annealed mixture. The reaction mixture was incubated at 45 °C for 30 min. Then, 5 μL of mixture containing 1 U SplintR ligase and 10% PEG 6000 was added to 15 μL of the elongated mixture and incubated at 37 °C for 30 min and 80 °C for 20 min and kept at 4 °C. qPCR was then performed using TB Green^®^ Premix Ex Taq™ II (Takara) according to the manufacturer’s protocol. Primers used in the SELECT assay are listed in Table S4.

### 2.8. Statistical Analysis

All statistical analyses, with the exception of the identification of differentially expressed genes (DEGs) in the microarray analysis, were performed using SPSS Statistics version 23.0.0 (IBM, Armonk, NY, USA). The DEGs in the microarray dataset were analyzed using the GEO2R tool. Adjusted *p* values were calculated using the Benjamini–Hochberg false discovery rate (FDR) procedure. For pairwise comparison of each experimental group, a *t*-test was performed. For comparisons involving more than three experimental groups, one-way ANOVA was performed, followed by Dunnett’s test or Tukey HSD as a post hoc analysis. To confirm the correlation between genes, Pearson or Spearman correlation tests were performed as appropriate. *p* values < 0.05 were considered statistically significant.

## 3. Results

### 3.1. Increased Expression of ADAM32 Under Hypoxic Conditions

**Figure 1:**
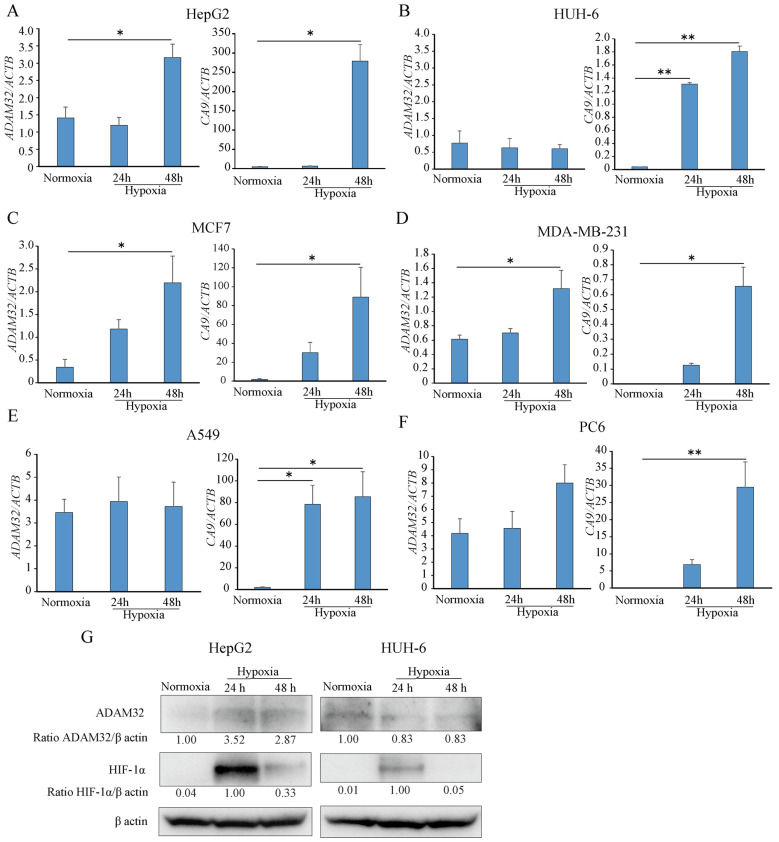
ADAM32 is increased under hypoxic conditions. Hepatoblastoma cell line, HepG2 (**A**), HUH-6 (**B**), breast cancer cell line, MCF7 (**C**), MDA-MB-231 (**D**), lung cancer cell line, A549 (**E**), and PC6 (**F**) were exposed to 1% O_2_ (hypoxia) for 24 and 48 h. The expression levels of a disintegrin and metalloprotease domain 32 (*ADAM32*) and the hypoxia-inducible factor (HIF) target gene, carbonic anhydrase 9 (*CA9*), were then evaluated by real-time RT-PCR (*n* = 3; one-way ANOVA with Dunnett’s test). Relative mRNA levels were calculated as a ratio to *ACTB* levels. Values are expressed as mean ± SE; * *p* < 0.05; ** *p* < 0.01. (**G**) Immunoblotting was performed using whole cell extracts from HepG2 and HUH-6 cells from each experiment. Representative blots from more than three independent experiments are shown. Relative expression levels of ADAM32 and HIF-1α were calculated using β-actin expression as the denominator for each sample. The uncropped bolts are shown in Figure S10.

In order to elucidate the regulatory mechanisms of *ADAM32* under hypoxic conditions, the HBL cell line as well as breast and lung cancer cell lines were utilized. When exposed to 1% O_2_ (hypoxia), the expression levels of *ADAM32* and carbonic anhydrase 9 (*CA9*), a known HIF-targeted gene, increased after 48 h in HepG2, MCF7, and MDA-MB-231 compared to those of 20% O_2_ (normoxia) (Figure 1A,C,D). On the other hand, expression levels of *ADAM32* remained unchanged in HUH-6 and lung cancer cell lines, although an increase in *CA9* expression was observed (Figure 1B,E,F). The expression levels of *ADAM32* in HepG2 and MCF7 did not change at any time point under normoxic conditions (Figure S3). Subsequently, the expression levels of ADAM32 and HIF-1α were evaluated by immunoblotting. The level of HIF-1α started to increase at 24 h and decreased at 48 h in HepG2 and HUH-6. Conversely, the protein expression of ADAM32 exhibited an initial increase at 24 h in HepG2, whereas it remained constant in HUH-6 (Figure 1G).

### 3.2. Increased ADAM32 Expression Under Hypoxic Conditions Is Regulated by HIF-1α but Not Promoter Regulation in HBL

**Figure 2:**
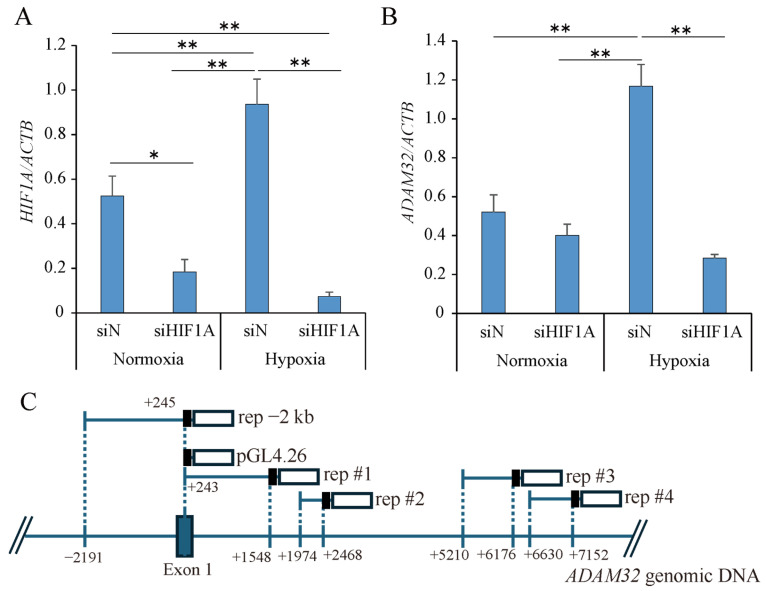

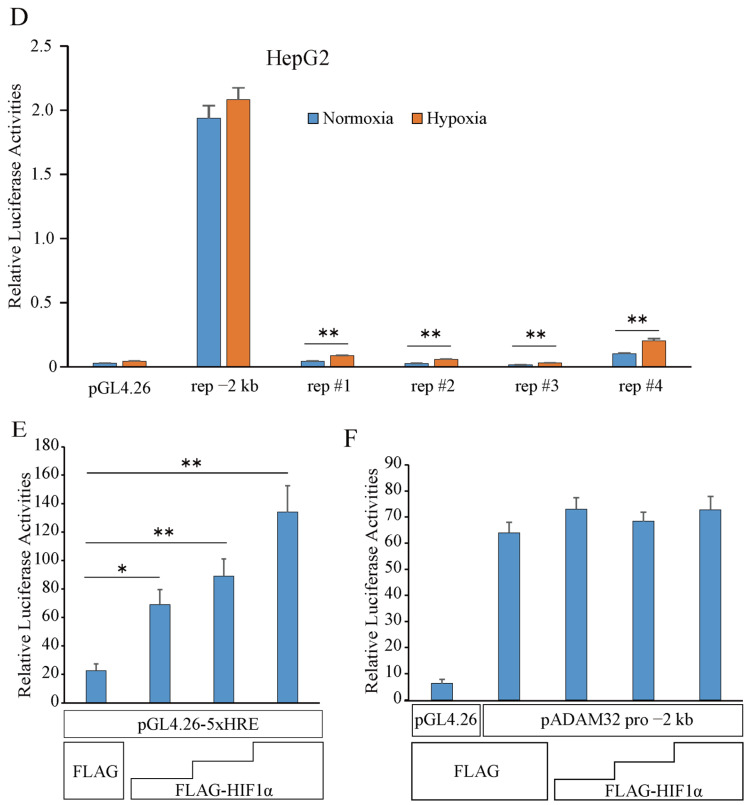
Increased *ADAM32* under hypoxic conditions is regulated by HIF-1α but not by promoter regulation in HBL. (**A**,**B**) HepG2 was transfected with siN and siHIF1A and then exposed to hypoxia for 48 h. The expression levels of *HIF1A* and *ADAM32* were evaluated by real-time RT-PCR (*n* = 5; one-way ANOVA with Tukey HSD). Relative mRNA levels were calculated as a ratio to *ACTB* levels. (**C**) Five promoters within the regions −2191 to +245 (rep −2 kb), +243 to +1548 (rep #1), +1974 to +2468 (rep #2), +5210 to +6176 (rep #3), and +6630 to +7152 (rep #4) of *ADAM32* were subcloned into pGL4.26 to generate the luciferase reporter constructs. (**D**) HepG2 was transiently transfected with luciferase reporters of the *ADAM32* promoter and exposed to hypoxia for 48 h. The promoter activities of each reporter were then evaluated by a luminometer (*n* = 4; *t*-test). (**E**) HepG2 was co-transfected with pGL4.26 5xHRE and FLAG-HIF-1α (*n* = 5; one-way ANOVA with Dunnett’s test). (**F**) HepG2 was co-transfected with pGL4.26 ADAM32 pro −2 kb and FLAG-HIF-1α. Promoter activities are shown as above (*n* = 4; one-way ANOVA). Values are expressed as mean ± SE; * *p* < 0.05; ** *p* < 0.01.

To evaluate the relevance of HIF-1α for ADAM32 expression under hypoxic conditions, knockdown experiments were performed. The knockdown of *HIF1A* attenuated the expression levels of *ADAM32* under hypoxic conditions for 48 h in HepG2 (Figure 2A,B, Figures S4 and S12). For the purpose of evaluating the promoter activity of *ADAM32*, the promoter and its adjacent region of *ADAM32* were first observed, focusing on HRE motif, DNase I hypersensitivity site, and chromatin immunoprecipitation sequence results. Then, the five regions were selected as sites to be evaluated, and reporters were constructed (Figure 2C and Figure S1). We then confirmed whether their reporter activity could be enhanced by hypoxic treatment. Contrary to our expectation, the promoter activity of rep −2 kb did not change under hypoxic conditions compared to normoxia. The activities of rep #1, rep #2, rep #3, and rep #4 increased under hypoxic conditions, although these activities were much lower than those of rep −2 kb in HepG2 (Figure 2D). To further confirm the relevance of HIF-1α in the promoter activity of *ADAM32*, a promoter assay combined with transient transfection of HIF-1α was performed. In a control experiment, transient transfection of FLAG-HIF-1α enhanced HRE reporter activity in a dose-dependent manner. (Figure 2E). When FLAG-HIF-1α was overexpressed, the promoter activity of rep −2 kb did not increase in HepG2 (Figure 2F).

### 3.3. ADAM32 Expression Under Hypoxic Conditions Is Modified by N^6^-Methyladenosine (m^6^A)-Related Regulation

**Figure 3:**
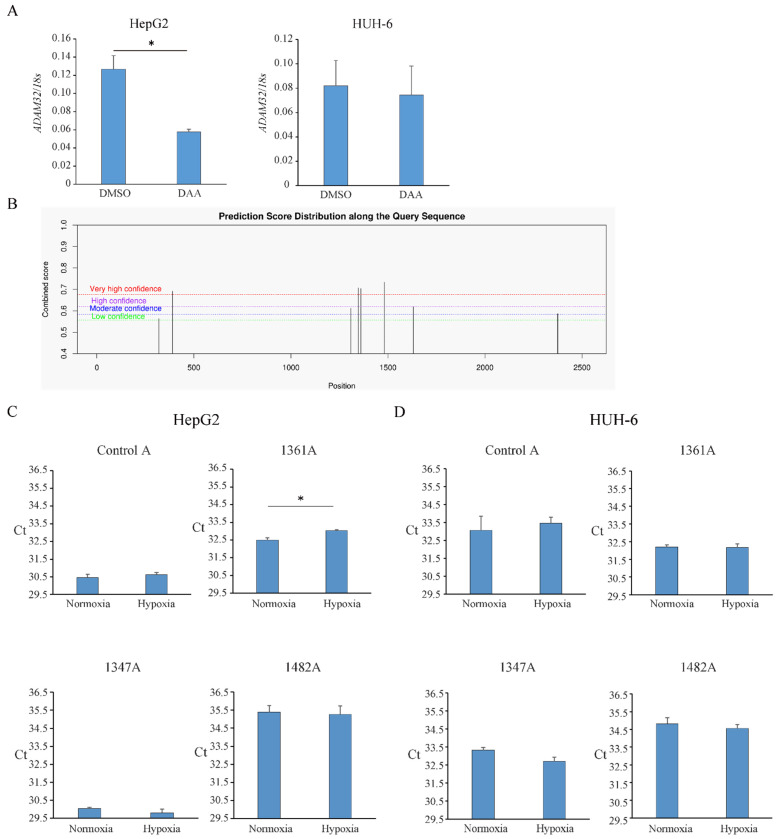
Increased *ADAM32* is regulated by an m^6^A mRNA-methylation-related mechanism. (**A**) HepG2 and HUH-6 were treated with 3-deazaadenosine (DAA) for 24 h. The expression levels of *ADAM32* were then evaluated by real-time RT-PCR (*n* = 3; *t*-test). (**B**) m^6^A sites were predicted by the sequence-based RNA adenosine methylation site predictor (SRAMP). (**C**,**D**) HepG2 and HUH-6 were exposed to hypoxia for 24 h. The m^6^A levels of Control A, 1361A, 1347A, and 1482A in these cells were then evaluated by a single-base elongation- and ligation-based qPCR amplification method (SELECT) (*n* = 4; *t*-test). Relative mRNA levels were calculated as the ratio to *18S* levels. Values are expressed as mean ± SE. * *p* < 0.05.

It has been reported that RNA expression is regulated by an m^6^A mRNA-methylation-related mechanism. To confirm the relevance of m^6^A mRNA methylation in *ADAM32* mRNA expression, 3-deazaadenosine (DAA)(Cayman Chemical, Ann Arbor, MI, USA), an inhibitor of m^6^A mRNA methylation, was used. The concentration of DAA was determined by preliminary experiments (Figure S5). DAA (10 μM) treatment decreased the expression level of *ADAM32* in HepG2, while that in HUH-6 remained unchanged (Figure 3A). In addition, we tried to confirm the level of m^6^A mRNA methylation under hypoxic conditions. First, m^6^A sites were predicted by SRAMP. We found that some possible m^6^A sites containing the consensus motif (DRACH) were predicted with high confidence scores (Figure 3B and Figure S6 and Table S4). Then, the levels of m^6^A at these sites were confirmed by the SELECT assay. The levels of the control sites at 1329 nt (Control A), m^6^A at 1347 nt (1347A), and 1482 nt (1482A) remained unchanged, while the level of m^6^A at 1361 nt (1361A) increased under hypoxic conditions in HepG2 but not in HUH-6 (Figure 3C,D).

### 3.4. ADAM32 Is Regulated by HIF1α/IGF2BP2 Signal

**Figure 4:**
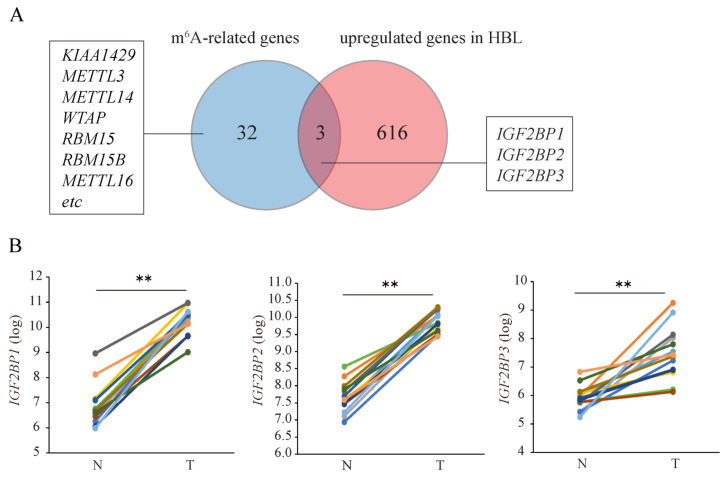

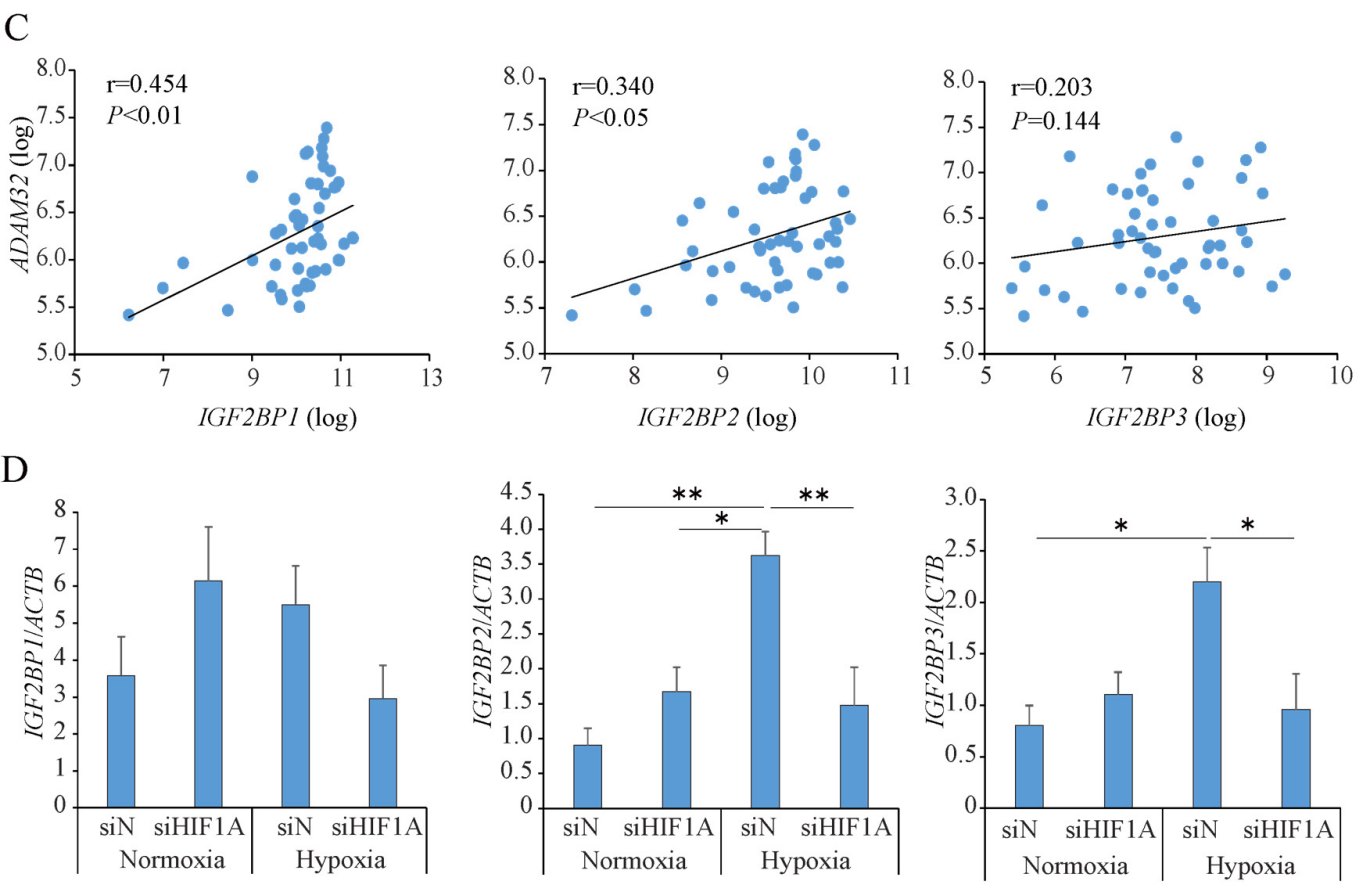
*ADAM32* is regulated by HIF1α/IGF2BP2 signaling. (**A**) The Venn diagram shows that 619 genes (right circle) in hepatoblastoma (HBL) tumor were 2-fold higher than in non-cancerous liver tissue, and 35 genes (left circle) are listed as m^6^A-related gene. Insulin-like growth factor 2 mRNA binding protein 1 (*IGF2BP1*), insulin-like growth factor 2 mRNA binding protein 2 (*IGF2BP2*), and insulin-like growth factor 2 mRNA binding protein 3 (*IGF2BP3*) are common genes in these conditions. (**B**) The expression levels of *IGF2BPs* in microarray data of paired samples N (non-cancerous liver) and T (tumor) are shown. Each paired sample is denoted by a unique colored line. (*n* = 14; paired *t*-test). (**C**) The correlation between the expression levels of *ADAM32* and *IGF2BP1* (Spearman correlation test), *IGF2BP2* (Spearman correlation test), *IGF2BP3* (Pearson correlation test) in the microarray data are shown (*n* = 53). (**D**) HepG2 was transfected with siN and siHIF1A, then these cells were exposed to hypoxia for 48 h. The expression levels of *IGF2BPs* were then evaluated by real-time RT-PCR. Relative mRNA levels were calculated as a ratio to *ACTB* levels (n = 5; one-way ANOVA with Tukey HSD). Values are expressed as mean ± SE; * *p* < 0.05; ** *p* < 0.01.

In the experiments, the m^6^A was inhibited by its inhibitor, and the m^6^A level of *ADAM32* increased under hypoxic conditions in the HBL line, HepG2. These findings suggest that the expression of *ADAM32* in HBL is modulated by a mechanism related to m^6^A. Therefore, we explored the molecules associated with *ADAM32* expression using microarray data of HBL. The 619 genes in HBL were 2-fold higher than in non-cancerous liver tissues (Figure 4A). The Venn diagram shows that the expression levels of insulin-like growth factor 2 mRNA binding protein 1 (*IGF2BP1*), *IGF2BP2* and insulin-like growth factor 2 mRNA binding protein 3 (*IGF2BP3*) were elevated in the tumor microarray data of HBL, and these genes were included in the m^6^A-related gene list (Figure 4A,B and Table S5) [28]. Furthermore, the expression levels of *ADAM32* correlated with those of *IGF2BP1* and *IGF2BP2* in these datasets (Figure 4C). Next, to clarify the relationship between HIF and m^6^A-related genes, we observed their levels in *HIF1A* knockdown experiments in HepG2. Real-time RT-PCR showed that the expression levels of *IGF2BP2* and *IGF2BP3* increased under hypoxic conditions and were decreased by *HIF1A* knockdown. In contrast, *IGF2BP1* expression remained unchanged between normoxia and hypoxia (Figure 4D). In addition, *ADAM32* mRNA was more stabilized under hypoxic conditions after actinomycin D treatment (Figure S7). Based on these results, we decided to focus on IGF2BP2.

### 3.5. Increased Expression of ADAM32 Is Regulated by IGF2BP2

**Figure 5:**
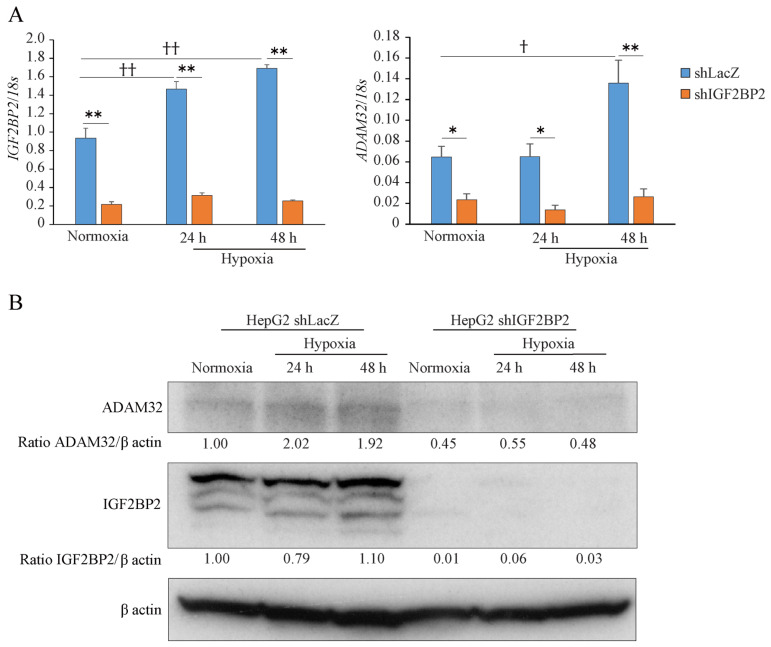
Increased expression of ADAM32 is regulated by IGF2BP2. (**A**) HepG2 shLacZ and shIGF2BP2 were exposed to normoxia or hypoxia for 24 and 48 h. The levels of *IGF2BP2* and *ADAM32* were then evaluated by real-time RT-PCR. Relative mRNA levels were calculated as the ratio to *18S* levels (*n* = 3). Values are expressed as mean ± SE; * *p* < 0.05 vs. shLacZ (*t*-test); ** *p* < 0.01 vs. shLacZ (*t*-test); † *p* < 0.05 vs. normoxia (one-way ANOVA with Dunnett’s test); †† *p* < 0.01 vs. normoxia (one-way ANOVA with Dunnett’s test). (**B**) Immunoblotting was performed using whole cell extracts from HepG2 shLacZ and HepG2 shIGF2BP2 cells under normoxic or hypoxic conditions for 24 and 48 h. Representative blots from more than three independent experiments are shown. Relative expression levels of ADAM32 and IGF2BP2 were calculated using β-actin expression as the denominator for each sample. The uncropped bolts are shown in Figure S11.

We further established stable knockdown cells of IGF2BP2 by shRNA transfection. Real-time RT-PCR showed that the expression levels of *IGF2BP2* and *ADAM32* were decreased in HepG2 shIGF2BP2 compared with that of HepG2 shLacZ under normoxic conditions. Under hypoxic conditions, the levels of *IGF2BP2* and *ADAM32* increased in HepG2 shLacZ. While the levels of *IGF2BP2* and *ADAM32* remained unchanged in HepG2 shIGF2BP2, these levels were much lower than in HepG2 shLacZ at all time points (Figure 5A). Immunoblotting showed that the expression of ADAM32 started to increase in HepG2 shLacZ at 24 h. On the other hand, the expressions of IGF2BP2 and ADAM32 decreased significantly in HepG2 shIGF2BP2 at all time points (Figure 5B).

## 4. Discussion

Recent studies have identified ADAM32 as a potential molecular target for the treatment of HBL due to its high expression levels in HBL [9]. However, the regulatory mechanisms underlying ADAM32 expression remain largely unexplored. Given that several ADAM family members are upregulated under hypoxic conditions in various cancers [13,14], we hypothesized that the tumor microenvironment plays a role in ADAM32 regulation. To investigate this, we examined ADAM32 expression under hypoxic conditions and found that while its levels increased in some cancer cell lines, including HBL, the response varied between different cell types. Notably, ADAM32 expression was increased in HepG2 cells derived from a poorly differentiated HBL sample [21] but remained unchanged in HUH-6 cells derived from a well differentiated HBL sample [22]. This suggests that intrinsic differences in tumor subtypes may influence ADAM32 regulation in response to hypoxia. Indeed, the expression of *CA9*, which is a HIF-targeted gene, was much more increased in HepG2 than in HUH-6 under hypoxic conditions, which might be due to the different HIF-related signaling (Figure 1A,B). HIF-related signaling might also contribute to the different response to hypoxia. To clarify these details, further studies such as RNAseq will be needed in future studies.

Given the well-established role of HIF-1α in mediating hypoxia-induced gene expression [15,16], we investigated its potential involvement in ADAM32 regulation. Consistent with previous findings, HIF-1α protein levels increased under hypoxic conditions in our study, suggesting a possible regulatory link. The expression peaked at 24 h and started to decrease at 48 h, which might have been because of the negative feedback mechanism [29]. Indeed, *HIF1A* knockdown resulted in the downregulation of *ADAM32* under hypoxic conditions, further supporting the role of HIF-1α in *ADAM32* expression. However, promoter activity assays showed that the −2 kb *ADAM32* promoter fragment had the highest activity, but there was no significant difference between normoxic and hypoxic conditions. Furthermore, the overexpression of HIF-1α did not increase promoter activity, suggesting that *ADAM32* upregulation under hypoxic conditions is primarily controlled by post-transcriptional mechanisms such as RNA modification rather than direct promoter activation by HIF-1α, although there is a possibility that other promoter regions may play a central role in the response to HIF-1α.

RNA modifications have emerged as critical regulators of gene expression, and among them, N^6^-methyladenosine (m^6^A) is a well-characterized epitranscriptomic modification that affects mRNA stability and translation [30,31]. Given that certain hypoxia-responsive genes are regulated by m^6^A methylation [32,33], we investigated whether *ADAM32* is subject to m^6^A modification. Bioinformatic analysis identified a consensus m^6^A motif within the *ADAM32* mRNA sequence, and treatment with an m^6^A inhibitor, DAA, resulted in decreased *ADAM32* expression, suggesting regulation by m^6^A-related mechanisms. Further analysis revealed an increase in m^6^A methylation at the 1361nt position of *ADAM32* mRNA under hypoxic conditions, strongly suggesting that hypoxia-driven *ADAM32* expression is mediated by m^6^A modification.

To elucidate the crosstalk between HIF-1α signaling and m^6^A modification, we examined m^6^A-related enzymes, including methylases, demethylases, and m^6^A reader proteins [34]. Microarray analysis revealed that *IGF2BP1* and *IGF2BP2*, both known to stabilize mRNA, were upregulated in HBL tumor samples, and their expression correlated with *ADAM32* levels. Importantly, *IGF2BP2* expression increased under hypoxic conditions, but this induction was abolished by *HIF1A* knockdown, suggesting that *IGF2BP2* is a downstream target of HIF-1α. Furthermore, HIF-1-targeted genes are correlated with *IGF2BP2*, suggesting that *IGF2BP2* may be regulated by HIF-1α in the microarray analysis (Figure S8). Consistent with these results, it has been reported that *IGF2BP2* is increased under hypoxic conditions [35]. Additional studies are needed to clarify its regulatory mechanism under hypoxic conditions. Functional assays confirmed that *IGF2BP2* knockdown reduced *ADAM32* expression and attenuated its hypoxia-induced upregulation, demonstrating that IGF2BP2 plays a pivotal role in *ADAM32* regulation under both normoxic and hypoxic conditions in HepG2 cells.

The therapeutic potential of IGF2BP2 has recently gained attention, with specific inhibitors showing promise in suppressing cancer progression [36,37,38]. In light of our findings, targeting IGF2BP2 may provide a novel therapeutic strategy for HBL by disrupting *ADAM32* expression. These novel agents targeting IGF2BP2 may contribute to the inhibition of cell growth, cell migration, and chemoresistance regulated by ADAM32 [9]. However, IGF2BP2 is ubiquitously expressed in adult tissues and plays a role in physiological processes such as embryonic development and metabolism [39,40]. Therefore, further experiments are needed to ensure these effects, safety, and drug delivery before proceeding to clinical trials.

## 5. Conclusions

To our knowledge, this is the first study to demonstrate that *ADAM32* is regulated by the m^6^A reader protein IGF2BP2 in HBL (Figure S9). These findings open new avenues for cancer treatment, and further investigation is warranted to unravel the precise molecular interactions that govern this regulatory axis. A deeper understanding of these mechanisms may ultimately lead to the development of innovative therapies targeting ADAM32 and IGF2BP2 in HBL.

## Data Availability

The data that support the findings of this study are openly available in the Gene Expression Omnibus as GSE131329.

## Supplementary Materials

The following supporting information can be downloaded at https://www.mdpi.com/article/10.3390/cancers17111772/s1, Figure S1: The detailed sequences of the reporters and the location of HRE; Figure S2: Levels of *ACTB* and *18S* under normoxic and hypoxic conditions; Figure S3: The expression levels of *ADAM32* at different time points in HepG2 and MCF7; Figure S4: The expression level of HIF1α by siRNA transfection; Figure S5: The preliminary experiment to determine DAA treatment conditions; Figure S6: The detailed position of the m^6^A consensus motif in *ADAM32* mRNA; Figure S7: *ADAM32* mRNA was more stabilized under hypoxic conditions; Figure S8: Correlation between the expression levels of between *IGF2BP2* and HIF1-targeted genes; Figure S9: Summary of hypothetical regulatory mechanisms of ADAM32 expression in HBL; Figure S10: Raw data of immunoblot from Figure 1G; Figure S11: Raw data of immunoblot from Figure 5B; Figure S12: Raw data of immunoblot from Figure S4; Table S1: shRNA target sequences; Table S2: Primer and probe sets for real-time RT-PCR; Table S3: m^6^A prediction of *ADAM32* mRNA by SRAMP; Table S4: Primer for SELECT assay; Table S5: The list of m^6^A related genes.
