# Supplementary material for: ADAM32 Oncogene in Hepatoblastoma Is Regulated by IGF2BP2
Source: Cancers (Basel). 2025 May 26;17(11):1772. doi: 10.3390/cancers17111772 (PMC12153665; doi:10.3390/cancers17111772)
Supplement: Supplementary file 1 [file cancers-17-01772-s001.zip › cancers-3599220-supplementary.pdf]

Supporting information

Supplemental Material and Methods

To observe the promoter region of *ADAM32* gene

To observe the promoter region of *ADAM32* gene, UCSC Genome Browser (https://genome.ucsc.edu/cgi-bin/hgGateway) was used. To detect the transcriptional factor binding site, the human reference sequence (GRCh37) was used to display clusters of the DNase I hypersensitive sites and transcription factor ChIP-seq from ENCODE. The sequences of these regions were then obtained, and the consensus of HIF response element (HRE) was searched.

A

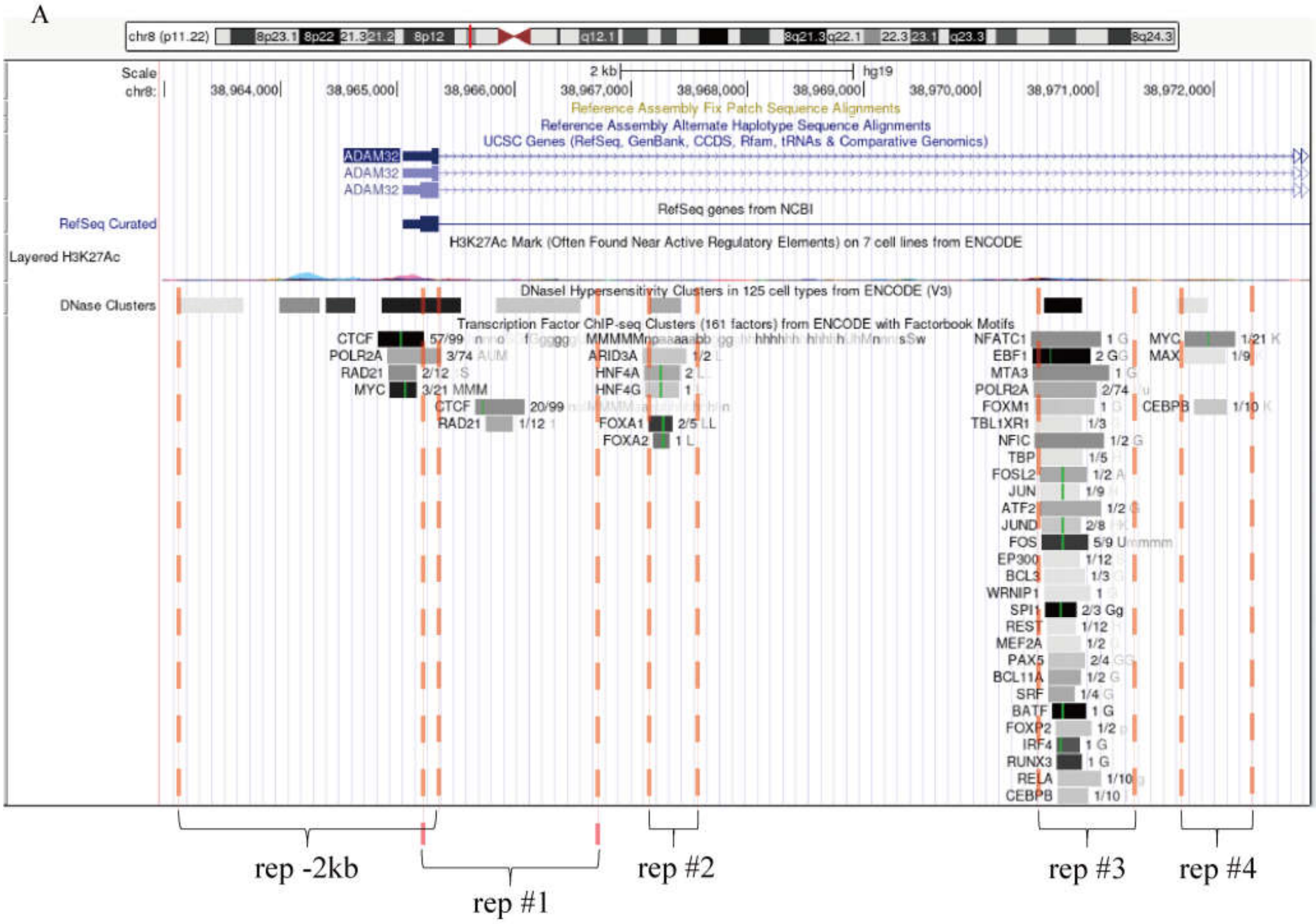

B

rep -2kb (-2191 to +245)

-2191
TTTAAATCTCTAAATGAATTCATATAAGACTCAAACGTTTGTGCTGTTCACTCATGTAGCCTCAGTTTTTGCAATTGTGATGCATATCACTGAAATTTTAC
AGTTGGTAATGTATTAACTATGTAACTTAACACTTGGAATTAAATAGTTCTTTGGATATTTGACTAGTTAAGTTTTTAAAAAGACTATTGGATCTGAACTT
CTTTTATATATTTTTTAATATGCTTTATGTTTTTTACTTACTTGGTCATGGATGCCACTTCAAAATTTGAAAATATTAAAGGACATCCTTTCTTTCTTCAAAA
AGAATAAATAAGGGTAAATTATATGCAAGTTTGACATCTCTGTTTGACTCTGTATGTCAAACTGACCTTGTTCAGAGTATTGCATTCTCGCTTTCTCCTGT
GTCTGTAACCATAGAGTTAAGCACAATTTGGGTTTTCTAAACTCAAATCAGATTTTATTAAATTTCATAAATGTTTACTAAAACCTACCATTTTTCAGGCA
CTGTATTAGGTACTGTGATATCTAAGTTCTGGATAGGATATGTTTCCCGACTTTTAGAAGTGCATCCTCTGTTATGAAGA GAGGCATACACACAAGAAT
AATACAAAATCAACCCTATTAAAGATACAAACCAGAAAGGTTGAGGAGTAAGAAAGAACAAAATAATACAAGAGCCAGAAATTCATTCAGAATACTTA
CCACTAATTGATGAGTTGAAGCATTTACTGTAATTGCTCTTCTGTTTGTTACAAAGCAAGGTGAAACTAGCTGTGGCTCAGCAGCTGCCTAAAAGCAGT
GTTAGCTTAGCATTGGCTGTTCCTATTGAGATACAAGCTCAGTGTATTTTTTTGTTGCCTTGTTCCCCACCCCACCCCTCCACCTGCCCCAGATGGAGTTT
TGCTCGTCACCCAGGCTGGAGGGCAATGGCACAATCTCGGCTCACTGCAACCTCCACTTCCTGGGCTCAAGCGATTCTCCTTCCTCAGCCTCCCCAAGT
GCTGGGATTACAGGCGCCCACAACCATGCCCGGCTAATTTTTGTATTTTTTAGTAGAAACGGGGTTTCACCATGTTGACCAGGCTGGTTTCAAACTCCT
HRE1
GACCTCTGGTGATCCAACCACCTCGGCCTCCCTAAGTGCTGGGATTACAGGCGTGAGCCACCTCACCTGGCCTGTTGCCTTGTTTTTGTTCATGCTATTA
HRE2
ATTTCAGGTGTAGATTGCCCTGTTGAAATTGCGTGCATCCATAAAAATCACATCTTCACTTTAAATGTCTTAGTTCCCCAAGTGAGATTTTTCTCTGTTCC
CATGAGGCCTTTTAGTTTATGTGTTGGCCTATTATGTAGTATGAGAAATTAATCCATTCTTTGGGATTTGAAAAGAATGAAAAGCTAATGCATACAGAT
GGAGACCAGGTGTGAGGCTGGGAAGGAGGAGGCATTGCAGTTCCAGGGAGTAAAGCAGTGTTTAGGAAAAACCCAAAGTCAGAGCTGCTAAAACAC
GGGATAGGAGAAATTAAGTGGCAGGTTCCGGTCTGAGTTCAGAAAAATTACTATGCTGAGGCTAGAATTTTCTACTGGAATGTAATTACTGTAATTGT
GGGCAAGAATCTCTAAATCTTTCACTACATGAAATAAGTATTTATTGCACTGAGAAGTTTCTGAACAATAGAATGACATCACATGAGTTTTAGCCAATG
GCCTGCGGAGAACCAGATTGGAGGAAAAAGCCTGGTTGTGAAGTCCATTTGGGCAGCGTCAGTGGGGAAAAAAATGCAGCTGGCGCTCTTCAAAAAT
ATGAAGAGCTGGCATTTCATATTGAAAAGCGGCTCACTTTTCCCTTCTTTGGAATGTTCACACATCTGGCTAAATAGTTTTGAAAAAATATTAGAAACAG
GTTTGGCAAATATGAATATATATTTAGGTGTGTATATAGATTTTAATTCAGAAAGTATTACACATTCGCTTTTGAAAGGACCTAGGAAGCCCCGAACGG
TGGCAGATGCCTGTAATCCCAGCTACTCAGGAAGTTGAGGAGGGAGGATCACTTGAACCCAGGAGTTCGAGACCAGCCTGGGCAGAATAGTGAGCAC
HRE3
CCCCGTCCCAAAAAGAAAAAAAAGCAACTGGGAAGTGTCTGGTGCAGGAGAGCACACGCTGACTGTGAATAAGTGTGTCAGTTCTTAAGGTCCAGCA
+1 HRE4
ACACAAAGCGAAAAGTTAGTGGAGGACTACGAGCGCGATCTCGACAGAGGGCGCTGGGTGGTCAGTGGCTCCAGCAACCACGCGGCTGGGGTGCGC
HRE5
CGGGAAGGGAGCTGGATGTTTTAGCCTCGGGGCGCACGCTGCGGGCCCTTCGTGTTCCGGACGCTAAACACCGAGAGCACCCCGTCTCCGGGGCCTC
HRE6
CGGAGAACGCTGTCCCATGAACGTGCGGGGAGCGGCCCCCGGCGTCCGCGCGTCCCCGCGTCCCTGGCAATTCCCGACTTCCCAACGGCTTCCCGCTG
+245
GCAGCCCCGAAGCCGCACCATGTTCCGCCTCTGGTTGCTGCTGGCCGGGCTCTGCGGCCTCCTGGCGTCAAGACCCGGTGAGCCAGCCCAGACCCTGA

C

rep #1 (+243 to +1548)

+243

GCAATTCCCGACTTCCCAACGGCTTCCCGCTGGCAGCCCCGAAGCCGCACCATGTTCCGCCTCTGGTTGCTGCTGGCCGGGCTCTGCGGCCTCCTGGCG
TCAAGACCCGGTGAGCCAGCCCAGACCCTGACACTAGTCCGGGCGCTCGTCACACTGCGGCCCGACTCCCTGCAAAGCCCGGGGCCCTCCCTGTCTGG
GTCCCTTTGGTCCTGTCACCCTGGCAACGGGGCCTTTTCCAGGGGATTAGGCGCCCCGTCCGGATGGAGAAGCGACTCAGGGCCCAGCACCAGGGCT
HRE1
CACGCCTGTAATCCCAGGGCTTTGAGAGACTGAGGCGAGCTGATTGTTTGACCTCGGAGTTGGAGACCAGCTTGGGCAACACAGCAAGACCCCATCTC
HRE2
TACAAAAAAATTTAAAAAAATGTTCCGAGGTGGTGGTGCGCACCTGTGATCCCAGCTACCCGGGAGGCTGAGGGAGGAGGACCACGTGAGTCCAGG
AGGTGGAGGCTGCAGTGAGCCAAGATCGCCCCCACTGGACTCCAGCCTGGGCGACAGAGATCCTGTCTTTTTTATAAATTAAAAAAATTATTTTTGTGT
GTGACCGAGTCCCTTCTGTCGCCCAGGCTGGAGTGCAGTGGGCAACAGTAGGCAACAGAGGGAGACTCCAGATCGTAGTTCTCCAGAACTATGAGGA
AAATAAATTTCTGTTGTTTAAGCCATGCAGTCTACACTATTTTGTTATGGCAGTGGGAGCTGACTAATATAGTACTTAACAGCAAAATGATTGAAAAACT
GTAAAAGTTCATTAGTAAGAGAACTGCTAAATAAACTGTGGTTATCTCCAAACAATGCAAATGGACCACTGTGGAGAGGGAGGAAGTAAGACATGCC
ATGTACTGACTCTGGCTGACAAGATTCAGAATAGTATGTAATAAGAGAAGGAAAAAAATACGGGCATATCCAAACATTTTACTTGCATGTAAGCCTCCC
TGGTGATACTGTAAATTTTTGAGAACTCTTCCTACTCTTCTTCCTATCCTGCCATAATTACCTCTGCAGCATAGCAGAGGTGTGGTCAAGGCCAGGGTGT
CAGCCTGGTTAGATTAGGGTGAGTGTTCTCTTCCTGGCTTGTAAACAGCTACCTTCTCCCTGCGTACTCACAGGACCTTTTCTTTGTGTACAGGGAAAGA
CAGGAGGAGGAAATTCTCTGATGTCTCATCTTATAAGGGCATGAATCTCATCATGAGGGTCCCACTCTATGCCTAAAACCCTGATTACCTCCCAAAGGC
CATAACCAAATACTGTCACATTGGGGGTTAGGGATTCAACATAGAATTTTGTGGGATACATTCAGTTCATAACACATACAAAATTTTAATGCAGTGGTT

+1548

D

rep #2 (+1974 to +2468)

+1947

AATCGGTTGAACCCAGGAGGCAGGAGTTGCAGTGAGCTGAGATGGCACCATCGCACTCCAGCCTGGGTGACAAGAGCAAAACTCCATCTCAAAAAAT
AAAATAAAAAAAAAAATATTAATGAACATTATTTTTTAGAGTACGTTTAGGTTCATAGCAAAATTGAGCTGAAAGAGTTCCCATATAGCCCCTGATCCCA
AATATGAACAGCTTCTCCCACTATCAACATCTGGCACCATGGGATCCATTTGTTACAATTGAGCCTACACTGACACATTGTTATCATCCAAAGTCCACAG
TTTGTTTGCATTAGGGTTCATTCTCCACATTGCCCATTCTATGGTTTTGACAAATGTACAATGACATGCATCCACCATTGTAGTATTATACAGAATAGTTT
CACTGCCCTACAAATCCCATGTGTTCTGCTTATTTATCCCTCCCTCCCCACTGCCCCTGGCAACAACTCATCTCCTTAGTCTTCTTAGTTTTGCCTTTTCCAG
AGTGTCATGTATTTGAAATCATATGTAGCCATTTTGGATGGACTTTTTTTTTACTTAATAATATGCATTTAAGGTTCTCCCATGCCTTTTCATGGCTTGATA

+2468

E

rep #3 (+5210 to +6176)

+5210

ACACAGGTAGGTCTCACTTAGAGACCTGTCATGAGACTGAGGATACACTGAAAGGGTTTAAATAAAGAGAGTTTAGCTATGGGACTATCTATAGAGAT
GTGGGCATCAATAATTAACGATGAAACGCACAGACTAGTAACAGTAGAAAGCCATATCACATGTGTTGAATTGATTGTTTGCAAAGATGACAATGGCC
TTAACACTTCCTCCATCCCTGTGTGTGCTTCCATTTTGCAACATTTCTTTGTCATTCCTCCCATTGAGAGGTAGAATGTATTTCTTCACTCTCTGAATCTGG
TTGGCCTCATTACTTGCTCTGGCCATTTGAATGTGGTAGAAATGAGTTGTGAGACTTCCAAGTCTATGCCTCAAGAGGCTTTGCAGCTTCCTCTCTGCTC
TCTTGAAGCACTGTCTTGACTGCTACATGAAGAATCCTAGACTAGGACGATAAGAGGATGTGTGAAACAGAGATGACTCAACCTAGGCCGTTCAGTCA
AAAGACATGAATGGGGTCGTCTTAGTCTCTCCAGGCCTAGGCGAGCTGCCAGATGACTGCAGCCACATGAGTAACATTATGAGTGTCAGTGCAGTGG
GTACTGCAGGGTGTCCTGTGTCATAACACTGAAGCAGGGCAGTGCCATCTATATCTTGAACTTGCTGCAGATTCTTCTCTTGCGCTGGGTCCAAGTCAA
TTTTGACAGTTTTACCAGTCACCCGATGAATGATTCAGGCTCTCACACCCCAAAACAGTATGTATTATCTCTGAATTCAAAAACTCAAAACGGCCTGCCA
CTCGTTGTGACTCTTTTTTTTTGTGGTGGACTATGCAAGGTGTAGCCACTAGCATCTCACTTTGTACAGGATATCTCAGCATGCTGCAACCACTGGACCC
CAAGAAACTTTGGGGTTGTAACTATCCCTTAACCTCCATAGGTTCTTCTACCACATTCTGGCATGCATGTATCTTACTGAAACATCTAGGGCACTTGCTAC

+6176

F

rep #4 (+6630 to +7152)

+6630

ACAATTTATCCCTTGATCTATATAAGTCCCTATTCTGATCAATGGACCATGCTGGTATTTTGAGTCCTCAGGATTAGTGTCAGTTCAGAGTCAGTAAATA
ATACTCCCTGAAAGTCTGGCTATTTCCCCTCCCCTGGGCATAGTCATCATGGTAAAAGGACACAGGACACTATGGAGGAGTCTGCAGGGGAAGATTCG
CAGCACATTCCTGTGGTTGCATGGCAAGGTCCTTCCTAAAGAGGACTCCCTCACTCCTTAATGAAAAGGCTGCAAGCCTCTGAACTGGGTCAGGTGTAG
GAACCAGGGAAGAGGCTGTGAGTCCTCGTGGTGATTGCATCAGTTTCTGCGCACCACAAGCTAGAATTTTCTTGACTAGACTGCTCATCTAGTTCATTC
CTAGGGACTCTGTGATCAGTTAGATACTACCAAGGATCTCTGTAGGTGAAAGCATTTTGTTTTTCACTCTGTCCTTGTGGCTTCTTCTGGTAATTGTGCTC
ACCTGTCTCTGAGAGTCAATTGTCTGCCACTTCAGAATTCCCTGATTCCCAGTCAAAGCAAGGAATCATGTTTAAAAATTTTTTTCATAAGTTTTATTTTA

+7152

Figure S1. The detailed sequences of the reporters and the location of HRE. (A) The clusters of the DNase I and transcription factor ChIP-seq are shown. (B-F) The sequence of each luciferase reporter is shown. And boxes indicate the positions of HRE in their reporters.

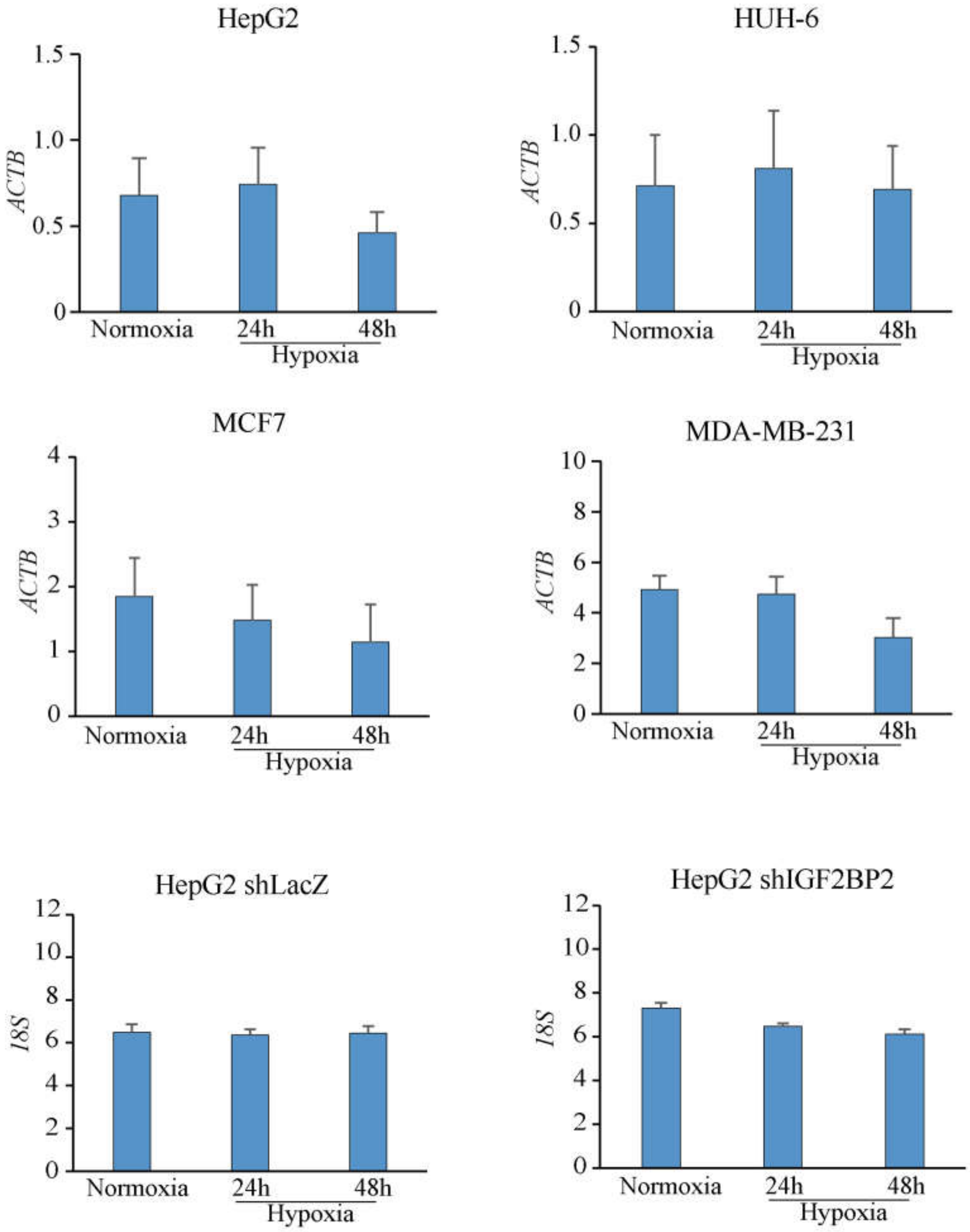

Figure S2. Levels of *ACTB* and *18S* under normoxia and hypoxia. HepG2, HUH-6, MCF7, MDA-MB-231, HepG2 shLacZ and HepG2 shIGF2BP2 were exposed to 1%O_2 (hypoxia) for 24, 48 hours. The levels of *ACTB* and *18S* were then evaluated by real-time RT-PCR. Values are expressed as mean ± SE (n = 3).

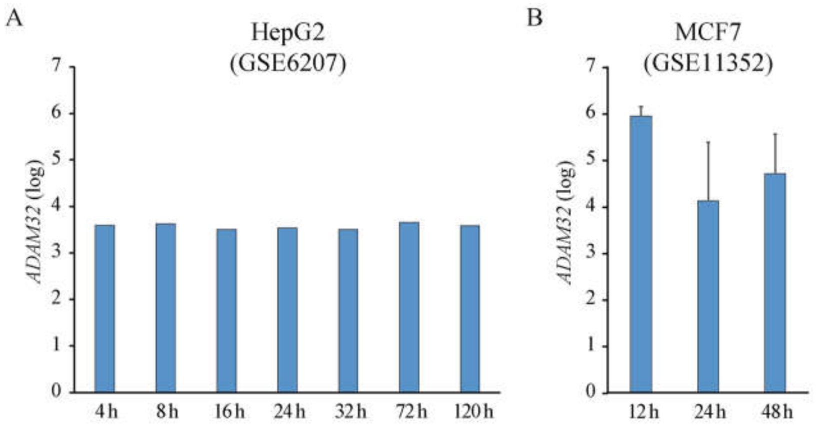

Figure S3. The expression levels of *ADAM32* at different time points in HepG2 and MCF7. (A) HepG2 cells were harvested at 4, 8, 16, 24, 32, 72, 120 hours after transfection of negative control miRNA duplex (GSE6207; n = 1). (B) MCF7 cells were harvested at 12, 24, 48 hours after vehicle treatment (GSE11352; n = 3). The expression levels of *ADAM32* were then evaluated by Affymetrix Human Genome U133 Plus 2.0 Array. Values are expressed as mean ± SE.

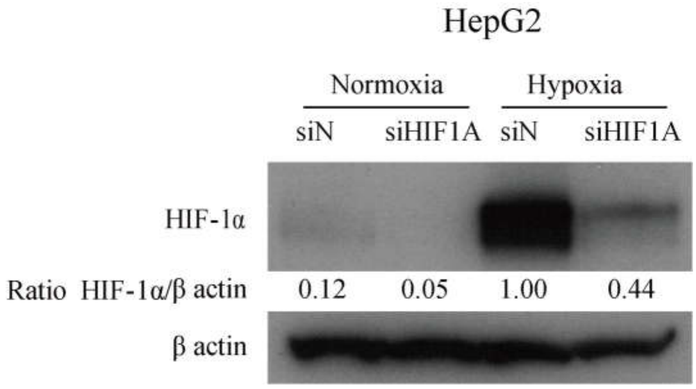

Figure S4. The expression level of HIF1α by siRNA transfection. HepG2 was transfected with siN and siHIF1A and then exposed to hypoxia for 48 hours. Immunoblotting was performed using whole cell extracts of these cells under normoxic or hypoxic conditions for 48 hours. Representative blots are shown. Relative expression levels of HIF-1α were calculated using β-actin expression as the denominator for each sample.

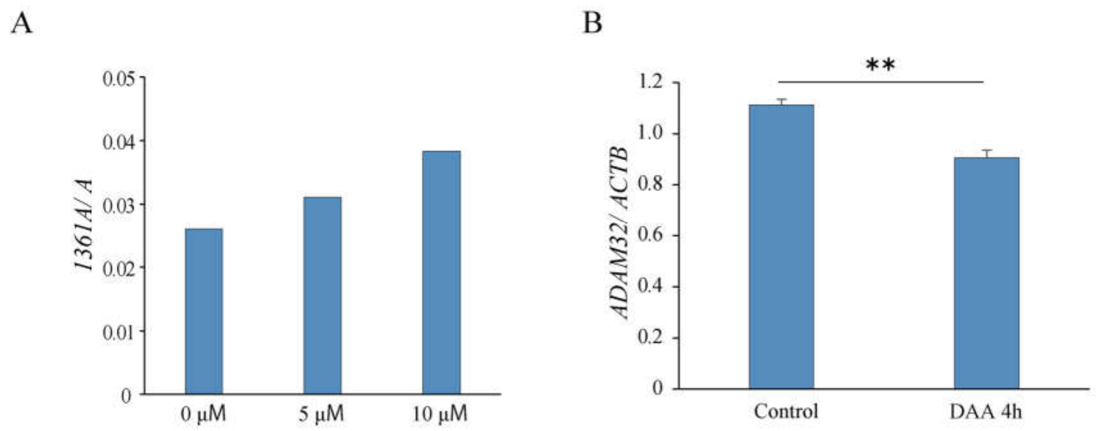

Figure S5. The preliminary experiment to determine the DAA treatment conditions. (A) HepG2 were treated with 3-Deazaadenosine (DAA) for 72 hours. Then, the level of control site at 1329 nt (Control A) and N6-methyladenosine (m^6A) levels at 1361nt (1361A) were evaluated by a single-base elongation- and ligation-based qPCR amplification method (SELECT). Relative 1361A levels were calculated as the ratio to control A levels ($n = 1$). The increased level of *1361A*/*A* indicates the decreased level of m^6A at 1361nt. (B) HepG2 were treated with DAA for 4 hours. The expression levels of *ADAM32* were then evaluated by real-time RT-PCR ($n = 3$; t test). Relative mRNA levels were calculated as the ratio to *ACTB* levels. Values are expressed as mean ± SE. $^{**}p < 0.01$.

1 *GACUUCCCAA CGGCUUCCCG CUGGCAGCCC CGAAGCCGCA CC*AUGUUCCG CCUCUGGUUG

61 CUGCUGGCCG GGCUCUGCGG CCUCCUGGCG UCAAGACCCG GUUUUCAAAA UUCACUUCUA

121 CAGAUCGUAA UUCCAGAGAA AAUCCAAACA AAUACAAAUG ACAGUUCAGA AAUAGAAUAU

181 GAACAAAUAU CCUAUAUUAU UCCAAUAGAU GAGAAACUGU ACACUGUGCA CCUUAAACAA

241 AGAUAUUUUU UAGCAGAUAA UUUUAUGAUC UAUUUGUACA AUCAAGGAUC UAUGAAUACU

301 UAUUCUUCAG AUAUUCAGAC UCAAUGCUAC UAUCAAGGAA AUAUUGAAGG AUAUCCAGAU

361 UCCAUGGUCA CACUCAGCAC GUGCUCUGGA CUAAGAGGAA UACUGCAAUU UGAAAAUGUU

421 UCUUAUGGAA UUGAGCCUCU GGAAUCUGCA GUUGAAUUUC AGCAUGUUCU UUACAAAUUA

481 AAGAAUGAAG ACAAUGAUAU UGCAAUUUUU AUUGACAGAA GCCUGAAAGA ACAACCAAUG

541 GAUGACAACA UUUUUAUAAG UGAAAAAUCA GAACCAGCUG UUCCAGAUUU AUUUCCUCUU

601 UAUCUAGAAA UGCAUAUUGU GGUGGACAAA ACUUUGUAUG AUUACUGGGG CUCUGAUAGC

661 AUGAUAGUAA CAAAUAAAGU CAUCGAAAUU GUUGGCCUUG CAAAUUCAAU GUUCACCCAA

721 UUUAAAGUUA CUAUUGUGCU GUCAUCAUUG GAGUUAUGGU CAGAUGAAAA UAAGAUUUCU

781 ACAGUUGGUG AGGCAGAUGA AUUAUUGCAA AAAUUUUUAG AAUGGAAACA AUCUUAUCUU

841 AACCUAAGGC CUCAUGAUAU UGCAUAUCUA CUAAUUUAUA UGGAUUAUCC UCGUUAUUUG

901 GGAGCAGUGU UUCCUGGAAC AAUGUGUAUU ACUCGUUAUU CUGCAGGAGU UGCAUUGUAC

961 CCCAAGGAGA UAACUCUGGA GGCAUUUGCA GUUAUUGUCA CCCAGAUGCU GGCACUCAGU

1021 CUGGGAAUAU CAUAUGACGA CCCAAAGAAA UGUCAAUGUU CAGAAUCCAC CUGUAUAAUG

1081 AAUCCAGAAG UUGUGCAAUC CAAUGGUGUG AAGACUUUUA GCAGUUGCAG UUUGAGGAGC

1141 UUUCAAAAUU UCAUUUCAAA UGUGGGUGUC AAAUGUCUUC AGAAUAAGCC ACAAAUGCAA

1201 AAAAAAUCUC CGAAACCAGU CUGUGGCAAU GGCAGAUUGG AGGGAAAUGA AAUCUGUGAU

1261 UGUGGUACUG AGGCUCAAUG UGGACCUGCA AGCUGUUGUG AUUUUCGAAC UUGUGUACUG

1321 AAAGACGGAG CAAAAUGUUA UAAAGGACUG UGCUGCAAAG ACUGUCAAAU UUUACAAUCA

1381 GGCGUUGAAU GUAGGCCGAA AGCACAUCCU GAAUGUGACA UCGCUGAAAA UUGUAAUGGA

1441 ACCUCACCAG AAUGUGGUCC UGACAUAACU UUAAUCAAUG GACUUUCAUG CAAAAAUAAU

1501 AAGUUUAUUU GUUAUGACGG AGACUGCCAU GAUCUCGAUG CACGUUGUGA GAGUGUAUUU

1561 GGAAAAGGUU CAAGAAAUGC UCCAUUUGCC UGCUAUGAAG AAAUACAAUC UCAAUCAGAC

1621 AGAUUUGGGA ACUGUGGUAG GGAUAGAAAU AACAAAUAUG UGUUCUGUGG AUGGAGGAAU

1681 CUUAUAUGUG GAAGAUUAGU UUGUACCUAC CCUACUCGAA AGCCUUUCCA UCAAGAAAAU

1741 GGUGAUGUGA UUUAUGCUUU CGUACGAGAU UCUGUAUGCA UAACUGUAGA CUACAAAUUG

1801 CCUCGAACAG UUCCAGAUCC ACUGGCUGUC AAAAAUGGCU CUCAGUGUGA UAUUGGGAGG

1861 GUUUGUGUAA AUCGUGAAUG UGUAGAAUCA AGGAUAAUUA AGGCUUCAGC ACAUGUUUGU

1921 UCACAACAGU GUUCUGGACA UGGAGUGUGU GAUUCCAGAA ACAAGUGCCA UUGUUCGCCA

1981 GGCUAUAAGC CUCCAAACUG CCAAAUACGU UCCAAAGGAU UUUCCAUAUU UCCUGAGGAA

2041 GAUAUGGGUU CAAUCAUGGA AAGAGCAUCU GGGAAGACUG AAAACACCUG GCUUCUAGGU

2101 UUCCUCAUUG CUCUUCCUAU UCUCAUUGUA ACAACCGCAA UAGUUUUGGC AAGGAAACAG

2161 UUGAAAAAGU GGUUCGCCAA GGAAGAGGAA UUCCCAAGUA GCGAAUCUAA AUCGGAAGGU

2221 AGCACACAGA CAUAUGCCAG CCAAUCCAGC UCAGAAGGCA GCACUCAGAC AUAUGCCAGC

2281 CAAACCAGAU CAGAAAGCAG CAGUCAAGCU GAUACUAGCA AAUCCAAAUC AGAAGAUAGU

2341 GCUGAAGCAU AUACUAGCAG AUCCAAAUCA CAGGACAGUA CCCAAACACA AAGCAGUAGU

2401 AACUAG*UGAU UCCUUCAGAA GGCAACGGAU AACAUCGAGA GUCUCGCUAA GAAAUGAAAA*

2461 *UUCUGUCUUU CCUUCCGUGG UCACAGCUGA AAGAAACAAU AAAUUGAGUG UGGAUCAAUU*

2521 *UGCA*

Figure S6. The detailed position of the m^{6}A consensus motif in *ADAM32* mRNA. The sequence of the mRNA including 5' UTR, CDS and 3'UTR of *ADAM32* is shown. The consensus motif (red box) and nucleotide positions (red) of m^{6}A predicted by SRAMP (sequence-based RNA adenosine methylation site predictor) are indicated.

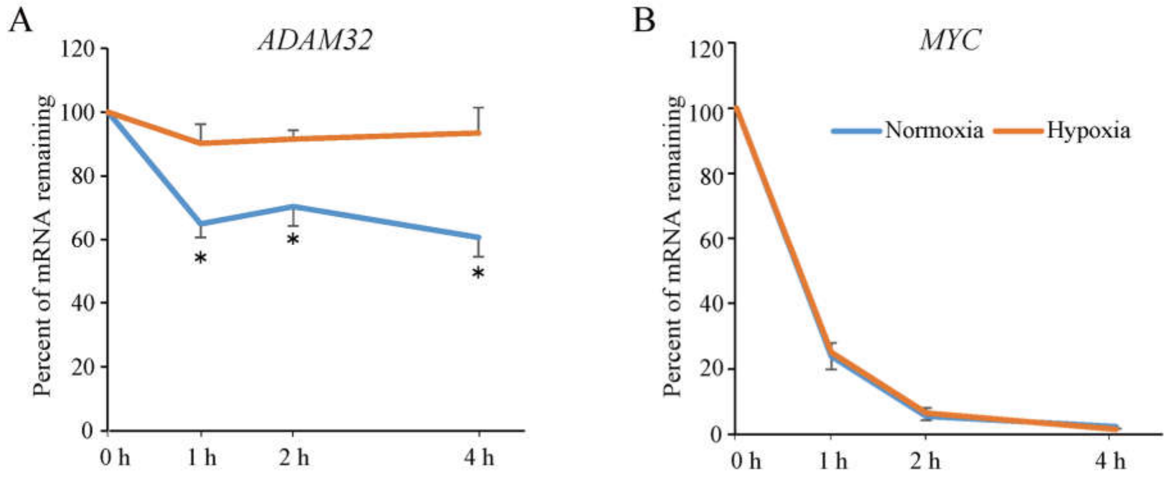

Figure S7. *ADAM32* mRNA was more stabilized under hypoxic conditions. HepG2 were treated with 5 µg/mL actinomycin D (Sigma, St. Louis, MO, USA) and then harvested at 0, 1, 2, and 4 hours. Levels of (A) *ADAM32* and (B) *MYC* proto-oncogene (*MYC*) were evaluated by real-time RT-PCR. The percentage of mRNA remaining at 1, 2, and 4 hours was then calculated. Values are expressed as mean ± SE ($n = 4$; t-test). * $p < 0.05$ vs. Hypoxia.

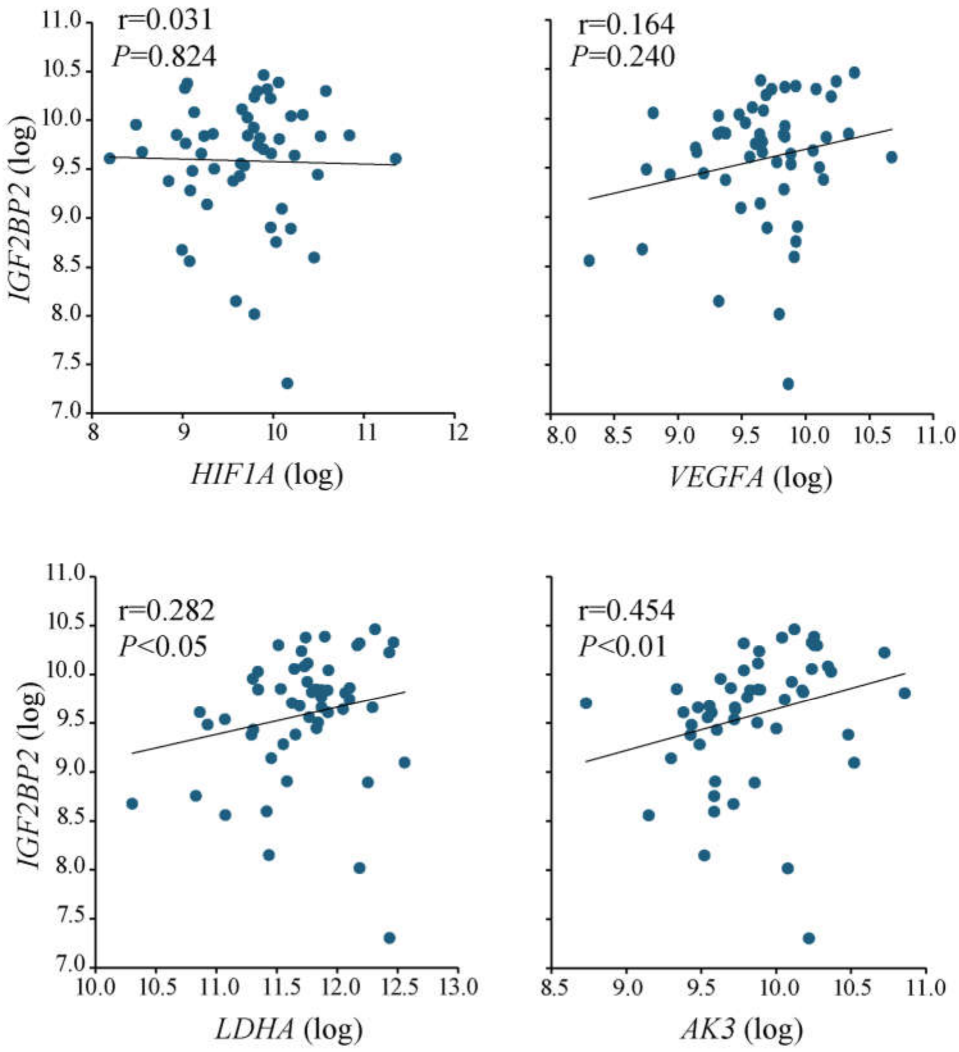

Figure S8. Correlation between the expression levels of *IGF2BP2* and HIF1-targeted genes. The correlation between the expression levels of insulin like growth factor 2 mRNA binding protein 2 (*IGF2BP2*) and hypoxia inducible factor 1 subunit alpha (*HIF1A*), vascular endothelial growth factor A (*VEGFA*), lactate dehydrogenase A (*LDHA*), and adenylate kinase 3 (*AK3*) in the microarray data (GSE131329) are shown (n = 53; Spearman correlation test).

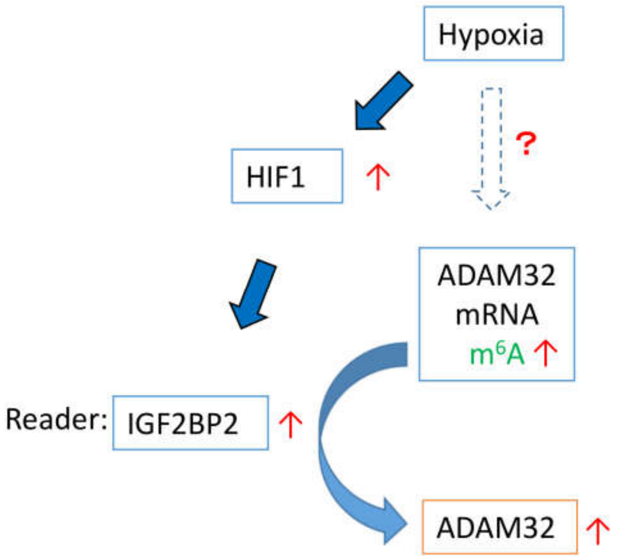

Figure S9. Summary of hypothetical regulatory mechanisms of ADAM32 expression in HBL. The expression of *ADAM32* is regulated by HIF-1α /IGF2BP2 signaling under both normoxic and hypoxic conditions.

Figure 1G

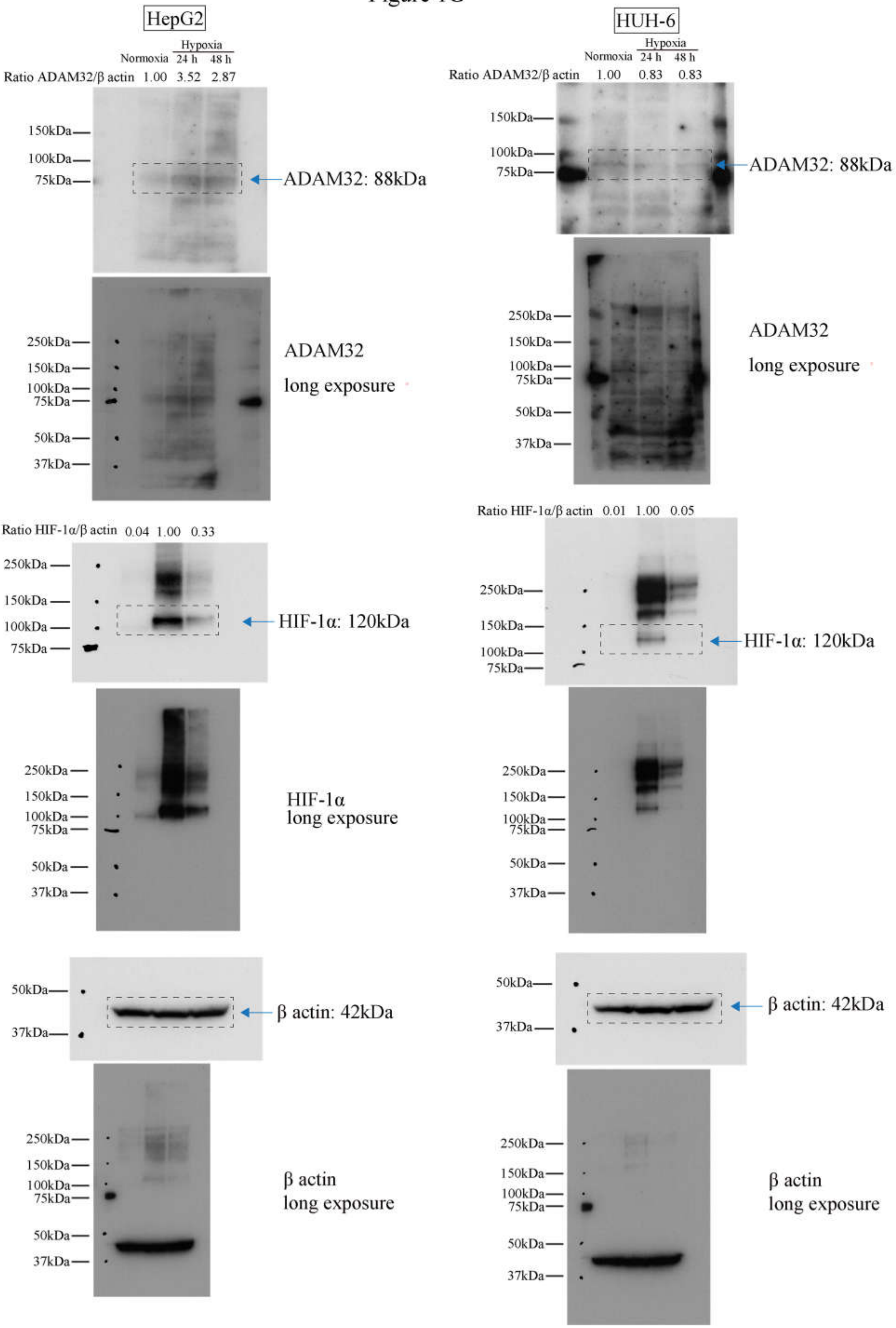

Figure S10. Raw data of immunoblot from Figure 1G.

Figure 5B

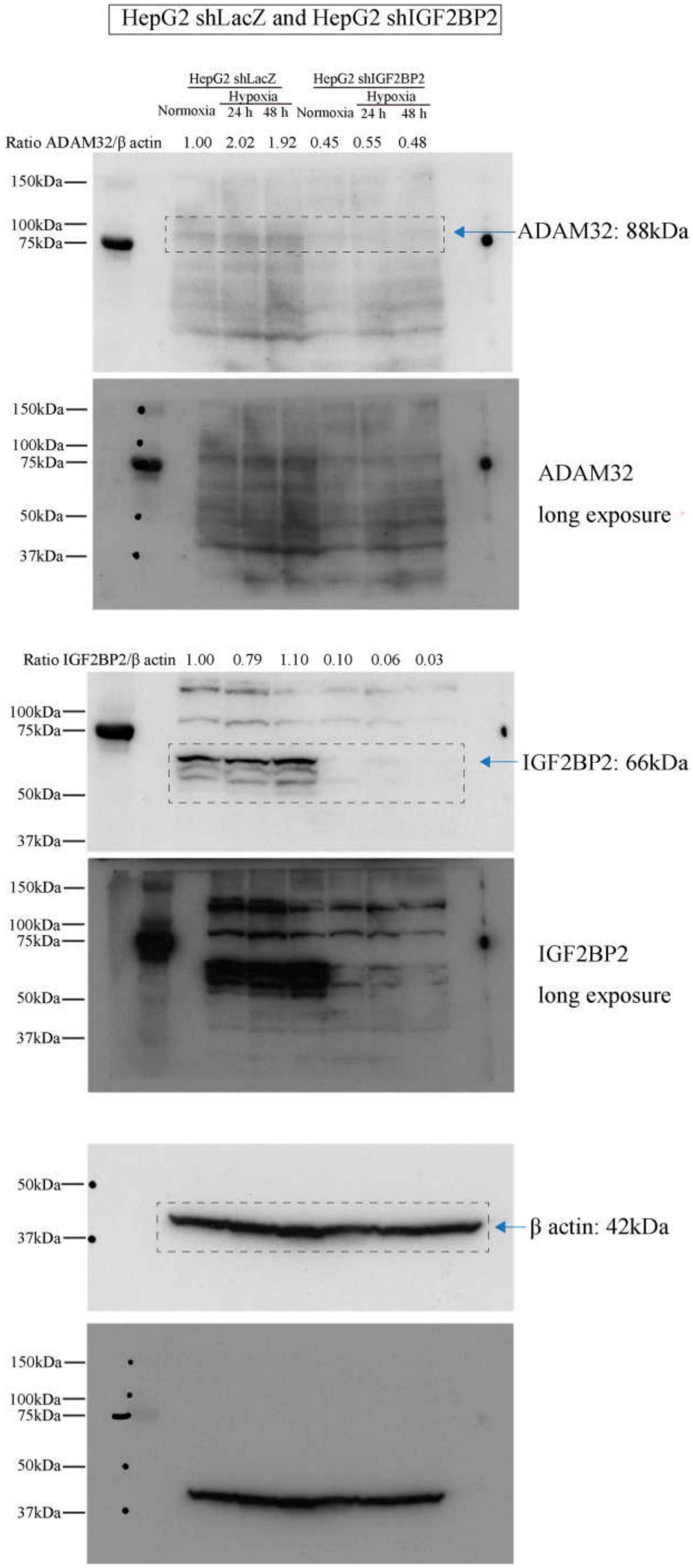

Figure S11. Raw data of immunoblot from Figure 5B.

Figure S4

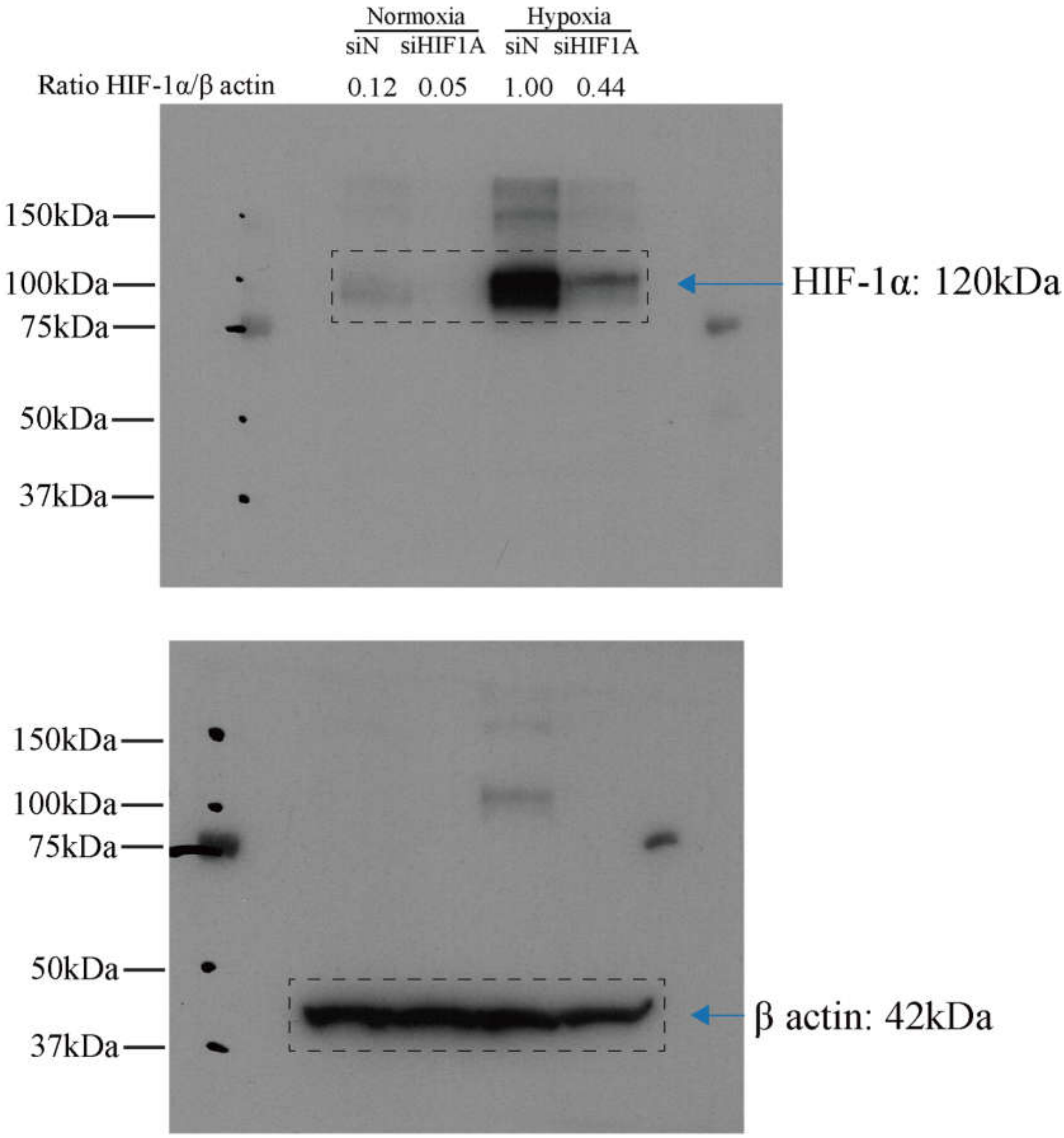

Figure S12. Raw data of immunoblot from Figure S4

Table S1. shRNA target sequences

Target sequence of shLacZ
5'- AGGTAAACAGTTGATTGAACTGC -3
Target sequence of shIGF2BP2
5'- TGGTGTATAAGCCATCAATATTC -3

Table S2. Primer and probe sets for real-time RT-PCR

ADAM32	Forward: 5'- ACCTAAGGCCTCATGATATTGC -3'<br>Reverse: 5'- TGCAAATGCCTCCAGAGTTA -3'<br>Universal Probe Library: #32
CA9	Forward: 5'- CCTTTGCCAGAGTTGACGAG -3'<br>Reverse: 5'- GCAACTGCTCATAGGCACTG-3'<br>Universal Probe Library: #25
HIF1A	Forward: 5'- GAACCTGATGCTTTAACTTTGCT -3'<br>Reverse: 5'- TGCTGGTCATCAGTTTCTGTG -3'<br>Universal Probe Library: #28
IGF2BP1	Forward: 5'- ACAAAACAGACCCAGTCCAAG -3'<br>Reverse: 5'- GCACACTGATGGCTTTTTCA -3'<br>Universal Probe Library: #12
IGF2PB2	Forward: 5'- CGTTCCCGCATCATCACT -3'<br>Reverse: 5'- CCCCTTCTTCCCGATGAT -3'<br>Universal Probe Library: #64
IGF2PB3	Forward: 5'- TCGGAACATCACCAAACAGA -3'<br>Reverse: 5'- GCAGAGGTGCCTTCAGGA -3'<br>Universal Probe Library: #66

Table S3. m6A prediction of *ADAM32* mRNA by SRAMP

#	Position	Sequence context	Score(binary)	Score(knn)	Score(spectrum)	Score(combined)	Decision
1	319	UACUUAUUCUUCAGAUAUUC**AGACU**CAAUGCUACUAUCAAGGAA	0.59	0.584	0.525	0.564	m6A site (Low confidence)
2	390	UCACACUCAGCACGUGCUCU**GGACU**AAGAGGAAUACUGCAAUUU	0.736	0.626	0.637	0.691	m6A site (Very high confidence)
3	1309	UGCAAGCUGUUGUGAUUUUC**GAACU**UGUGUACUGAAAGACGGAG	0.617	0.649	0.602	0.612	m6A site (Moderate confidence)
4	1347	ACGGAGCAAAAUGUUAUAAA**GGACU**GUGCUGCAAAGACUGUCAA	0.79	0.853	0.576	0.707	m6A site (Very high confidence)
5	1361	UAUAAAGGACUGUGCUGCAA**AGACU**GUCAAAUUUUACAAUCAGG	0.75	0.724	0.639	0.704	m6A site (Very high confidence)
6	1482	CUGACAUAACUUUAAUCAAU**GGACU**UUCAUGCAAAAAUAAUAAG	0.834	0.749	0.596	0.734	m6A site (Very high confidence)
7	1631	UCUCAAUCAGACAGAUUUGG**GAACU**GUGGUAGGGAUAGAAAUAA	0.702	0.673	0.499	0.619	m6A site (Moderate confidence)
8	2375	ACUAGCAGAUCCAAAUCACA**GGACA**GUACCCAAACACAAAGCAGU	0.682	0.699	0.44	0.586	m6A site (Moderate confidence)

Table S4. Primer for SELECT assay

ADAM32_A_1329 (Control A)
Up: 5'- TAGCCAGTACCGTAGTGCGTGCAGTCCTTTATAACATTTTGC -3'
Down: 5'- CCGTCTTTCAGTACACAAGCAGAGGCTGAGTCGCTGCAT -3'
ADAM32_m6A_1347 (1347A)
Up: 5'- TAGCCAGTACCGTAGTGCGTGACAGTCTTTGCAGCACAG -3'
Down: 5'- CCTTTATAACATTTTGCTCCGCAGAGGCTGAGTCGCTGCAT -3'
ADAM32_m6A_1361 (1361A)
Up: 5'- TAGCCAGTACCGTAGTGCGTGCCTGATTGTAAAATTTGACAG -3'
Down: 5'- CTTTGCAGCACAGTCCTTCAGAGGCTGAGTCGCTGCAT -3'
ADAM32_m6A_1482 (1482A)
Up: 5'- TAGCCAGTACCGTAGTGCGTGCTTATTATTTTTGCATGAAAG -3'
Down: 5'- CCATTGATTAAAGTTATGTCCAGAGGCTGAGTCGCTGCAT -3'
qPCR for SELECT
Forward: 5'- ATGCAGCGACTCAGCCTCTG -3'
Reverse: 5'- TAGCCAGTACCGTAGTGCGTG -3'

Table S5. The list of m^{6}A related genes

	m^{6}A related genes
Writers	KIAA1429, METTL3, METTL14, WTAP, RBM15,
	RBM15B, METTL16, ZC3H13, PCIF1
Readers	TRMT112, ZCCHC4, NUDT21, CPSF6, CBLL1
	SETD2, HNRNPC, HNRNPG, HNRNPA2B1
	IGF2BP1, IGF2BP2, IGF2BP3, YTHDC1, YTHDF1
	YTHDF2, YTHDF3, YTHDC2, SRSF3, SRSF10
	XRN1, FMR1, NXF1, PRRC2A
Erasers	FTO, ALKBH5, ALKBH3
